# Diversity, Community Assemblage, and Environmental Determinants of Phytoplankton in a Subtropical Transboundary Coastal River

**DOI:** 10.1002/ece3.72787

**Published:** 2025-12-23

**Authors:** Md. Saeduzzaman Faraji, Md. Mofizur Rahman, Md. Milon Sarker, Mehedi Mahmudul Hasan, Asma Jaman, Hea Ja Baek, Takaomi Arai, Norhayati Ngah, M. Belal Hossain

**Affiliations:** ^1^ Department of Fisheries and Marine Science Noakhali Science and Technology University Noakhali Bangladesh; ^2^ Department of Fisheries Management Bangladesh Agricultural University Mymensingh Bangladesh; ^3^ Bangladesh Fisheries Research Institute Mymensingh Bangladesh; ^4^ Department of Marine Biology Pukyong National University Busan Republic of Korea; ^5^ Environmental and Life Sciences Programme, Faculty of Science Universiti Brunei Darussalam Gadong Brunei Darussalam; ^6^ East Coast Environmental Research Institute Universiti Sultan Zainal Abidin Kuala Nerus Malaysia; ^7^ School of Engineering and Built Environment Griffith University Nathan Queensland Australia

**Keywords:** abundance and diversity, assemblage, community structure, Dakatia River, physicochemical parameters, phytoplankton

## Abstract

The health of subtropical transboundary coastal rivers is closely linked to phytoplankton diversity, seasonal fluctuations, and community structure. This study presents the first comprehensive multivariate assessment of phytoplankton diversity and environmental drivers in the Dakatia River. Canonical Correspondence Analysis (CCA), Analysis of Similarity (ANOSIM), and Similarity Percentage (SIMPER) were applied across eight geo‐referenced stations under varying anthropogenic pressures to establish a baseline data for this cage‐culture–intensive river system. The analysis revealed distinct spatial and seasonal variations in phytoplankton diversity and taxonomic composition, highlighting shifts in community assemblages and the influence of physicochemical factors on these dynamics. A total of 37 genera were identified across six major classes: Bacillariophyceae (36.50%), Chlorophyceae (25.91%), Cyanophyceae (17.00%), Euglenophyceae (13.20%), Ulvophyceae (5.06%), and Zygnematophyceae (2.31%). Phytoplankton abundance was highest in winter (16,958.33 ± 6418.75 cells L^−1^), followed by the monsoon (14,572.92 ± 2982.90 cells L^−1^) and summer (13,739.60 ± 1857.76 cells L^−1^). The Shannon–Wiener diversity index, species evenness, and species richness exhibited seasonal fluctuations, ranging from 2.14 ± 0.22 to 2.47 ± 0.15, 0.65 ± 0.07 to 0.75 ± 0.07, and 2.67 ± 0.55 to 4.29 ± 0.38, respectively, reflecting a moderate level of phytoplankton diversity in a dynamic and complex river ecosystem. Cluster analysis delineated five distinct phytoplankton communities at a 50% similarity threshold. ANOSIM results indicated significant seasonal dissimilarity (*p* < 0.001), while no significant spatial dissimilarity (*p* > 0.05) was observed. SIMPER analysis identified *Melosira* sp. as the primary contributor to interseasonal dissimilarities, followed by *Oscillatoria* sp. and *Gomphosphaeria* sp. Pearson's correlation and CCA highlighted ammonia, temperature, salinity, nitrate, and phosphate as key environmental drivers shaping phytoplankton abundance, diversity, and community composition. These findings have provided critical insights for ecosystem management, biodiversity conservation, and sustainable water resource governance.

## Graphical abstract

This study examined spatial and seasonal dynamics of phytoplankton communities in the Dakatia River, identifying 37 genera across six major taxonomic classes, with Bacillariophyceae being the most dominant. Seasonal peaks in phytoplankton abundance and diversity indices indicated higher richness and evenness during winter, while multivariate analyses revealed ammonia, temperature, salinity, nitrate, and phosphate as key environmental drivers. Statistical assessments showed significant seasonal variations in community structure (*p* < 0.001), with *Melosira* sp., *Oscillatoria* sp., and *Gomphosphaeria* sp. contributing most to interseasonal differences.

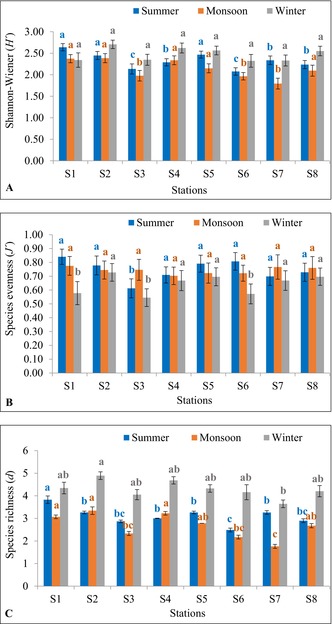

## 1 Introduction

Phytoplankton, as primary producers, are essential aquatic organisms that form the foundation of the aquatic food web, facilitating energy transfer to higher trophic levels (Mamun et al. 2019; Sharma et al. 2016). Although phytoplankton constitute only about 1% of the total biomass of Earth, they contribute roughly 90% of underwater primary production (Iqbal et al. 2017) and nearly 50% of global photosynthetic activity (Jahan and Singh 2023). Their key role in carbon fixation and oxygen production underlines their significance in sustaining aquatic ecosystems and regulating atmospheric composition. Nearly all aquatic organisms within a biological community rely on phytoplankton as their primary food source (Khan 2021; Falkowski et al. 2004), with zooplankton, finfish, and shellfish depending directly on them (Kumar et al. 2020; Suseela 2009). Natural food intake and the development of fish are directly connected with the quantity and quality of plankton available in aquatic bodies (Xavier et al. 2016; Rahman et al. 2008).

Diversity indices, including Shannon–Wiener diversity, species evenness, and species richness, are widely utilized to describe community structure and trophic status, serving as potential bioindicators for water quality assessment (Mamun et al. 2019; Telesh 2004). These indices are also fundamental for evaluating species diversity (Odum and Barrett 1971), community dynamics (Krebs 1989), and habitat suitability (Margalef 1968) in aquatic ecosystems. Furthermore, phytoplankton diversity indices provide critical insights into ecological status and habitat characteristics, supporting effective monitoring and management of aquatic ecosystems (Cardoso et al. 2012).

Phytoplankton diversity in aquatic ecosystems is closely linked to water physicochemical properties (Ahmed and Wanganeo 2015). Their sensitivity to environmental changes makes them reliable bioindicators of water quality (Esenowo et al. 2017; Medupin 2011) and key indicators of ecological interactions (Thakur et al. 2013). The spatial and temporal dynamics of phytoplankton are regulated by physicochemical factors that dictate their composition, abundance, and distribution, while seasonal fluctuations further influence these conditions, shaping community structure and diversity (Hasan et al. 2022). For example, monsoon precipitation and runoff increase turbidity and reduce light penetration, limiting photosynthesis and altering phytoplankton productivity and assemblages.

Rivers are among the most diverse and productive ecosystems, supporting a wide range of flora and fauna (Zhang et al. 2019; Marshall 2009; Zhou et al. 2009). In Bangladesh, rivers are integral to fishing, farming, navigation, and sanitation. The transboundary Dakatia River originates in the Tripura hills of India, enters Bangladesh through Cumilla, and eventually merges with the Meghna River (Hasan et al. 2018). It harbors over 70 fish species, including threatened ones, sustaining numerous livelihoods (Ahmed et al. 2021; Hasan et al. 2018; Hossain et al. 2017). Cage culture, a growing aquaculture practice, has expanded significantly in the Dakatia River, increasing from 40 cages in 2005 to 3510 in 2011 (Ahmed et al. 2014; Baqui and Bhujel 2011). However, industrial and municipal discharges have led to heavy metal contamination and low dissolved oxygen, threatening aquatic biodiversity (Hasan et al. 2015). Waste disposal from households, rice mills, and medical facilities further degrades water quality (The Financial Express 2022).

Previous studies have examined physicochemical properties and phytoplankton communities in several major rivers of Bangladesh, including the Meghna River (Hossain et al. 2016), the Halda River (Hossain, Akter, and Sarker 2022), the Karatoya River (Haque et al. 2021), the Padma River (Haque et al. 2019), and the Jamuna River (Akter et al. 2018). These investigations have provided insights into the composition, structure, and ecological dynamics of riverine phytoplankton. In contrast, the Dakatia River has not yet been comprehensively studied, despite its important role in supporting nearby fisheries and its increasing significance for cage aquaculture. The absence of baseline ecological data limits the assessment of river health and constrains the development of strategies for sustainable management of its fisheries. The diversity and assemblage patterns of phytoplankton, which are the building blocks of aquatic food webs, make them sensitive markers of nutrient status, water quality, and the general health of ecosystems. We hypothesize that anthropogenic impacts and geographical heterogeneity in physicochemical parameters significantly shape the phytoplankton community structure in the Dakatia River. This study intends to test this hypothesis by: (i) characterizing the diversity and community composition of phytoplankton in the Dakatia River; (ii) identifying seasonal and spatial variations in phytoplankton assemblages across various riverine zones; (iii) using multivariate analyses to identify the key environmental drivers regulating phytoplankton community structure; and (iv) providing crucial baseline data to guide the development of sustainable fisheries and aquaculture in this understudied coastal river system. The findings will contribute to informed management strategies, ensuring the ecological balance and economic viability of the riverine ecosystem.

## 2 Materials and Methods

### 2.1 Study Area and Sampling Procedure

**FIGURE 1:**
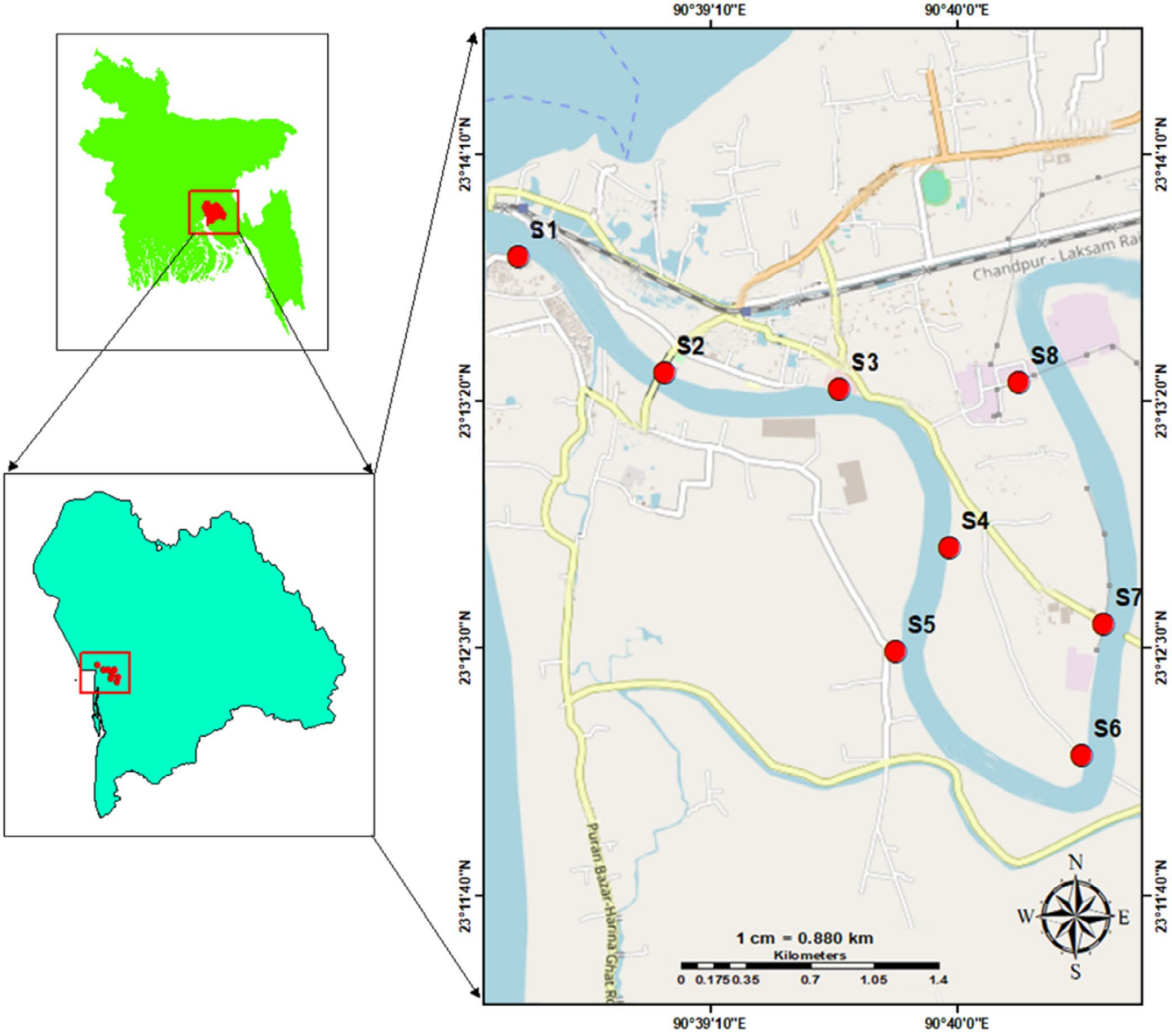
Location of the sampling stations of Dakatia River, Chandpur, Bangladesh.

The Dakatia River, located in the Chandpur district of Bangladesh, spans approximately 207 km in length and drains a catchment area of around 38.78 km^2^. The river has an average width of 180 m and an average depth of 9 m (Hasan et al. 2018). Phytoplankton samples were collected from eight geo‐referenced sampling stations along the river, situated between latitudes 23°12′ N and 23°13′ N and longitudes 90°38′ E and 90°40′ E (Figure 1). Stations S1 and S2, near the estuarine interface, experienced high sediment loads, strong currents, elevated salinity, and nutrient enrichment. S3 was dominated by dense aquatic vegetation influenced by domestic and industrial nutrient inputs, while S4 remained relatively pristine with minimal nutrient and occasional oil contamination. Cage culture zones S5 and S6 exhibited moderate flow, lower salinity, and localized organic pollution from feed and fish excreta. In contrast, S7 and S8 were exposed to severe anthropogenic pressures, including ship‐borne waste, oil spills, and industrial effluents containing heavy metals and other chemical contaminants, highlighting a clear gradient of ecological disturbance across the study area.

Seasonal sampling was conducted during three distinct periods—summer (April 2022), monsoon (July 2022), and winter (December 2022). At each station, triplicate samples were collected between 8:00 a.m. and 10:00 a.m. during each season to analyze phytoplankton community composition and associated physicochemical parameters.

### 2.2 Study of Physicochemical Parameters

Physicochemical parameters, including water temperature (°C), pH, dissolved oxygen (DO; mg L^−1^), electrical conductivity (μS cm^−1^), salinity (ppt), and total dissolved solids (TDS; mg L^−1^), were measured in situ using a multiparameter probe (Model HI98194, Hanna Instruments Inc., UK). Water transparency (cm) was assessed on‐site using a standard Secchi disk. For nutrient analyses, approximately 250 mL of water was collected from each site and filtered through Whatman GF/F glass fiber filters (pore size: 0.45 μm). Concentrations of nitrate (${\mathrm{NO}}_{3}^{-}$), phosphate (${\mathrm{PO}}_{4}^{3-}$), and ammonia (NH_3_) were determined ex situ using a portable spectrophotometer (Model DR2700, Hach Company, USA) following standard colorimetric procedures.

### 2.3 Sample Collection, Identification, and Enumeration

At each station, three separate surface samples (triplicates) were collected during each seasonal sampling event between 08:00 and 10:00. Approximately 40 L of surface water (integrated surface layer, ~0–0.5 m) from each replicate was passed through a 25 μm mesh plankton net and concentrated to a final volume of ~10 mL. Concentrates were preserved immediately in the field with 5% buffered formalin (final concentration), labeled and stored at 4°C until laboratory processing. In the laboratory, samples were gently homogenized prior to subsampling. For enumeration, a 1 mL aliquot of the homogenized concentrate was transferred to a Sedgwick–Rafter counting chamber (S–R), allowed to settle for at least 5 min, and examined under a Euromex EC 1152 light microscope at 100× and 400× magnifications. To reduce counting error, 10 randomly selected fields were counted per chamber; where sample heterogeneity was suspected, counts were repeated in a second chamber and averaged (i.e., typically 10–20 fields per sample). Phytoplankton density (cells L^−1^) was calculated following Sarker et al. (2021) as follows:

$$N=\frac{P\times C\times 1000}{L}$$

where *N* is the total amount of phytoplankton in 1 L of sample water, *P* is the average number of phytoplankton counted in 1 mL of concentrated water, *C* is the volume of the concentrated water (10 mL), and *L* is the total volume of the filtered water (40 L).

Taxonomic identification to genus level was made using standard taxonomic keys and published regional lists; identifications were cross‐checked by two experienced microscopists and ambiguous specimens photographed or mounted as voucher slides. Voucher preparations are archived at the Department of Fisheries and Marine Science, Noakhali Science and Technology University (NSTU) and are available on request.

### 2.4 Diversity Indices

To assess the diversity indices of phytoplankton, the Shannon–Wiener diversity index (*H′*) (Shannon and Weaver 1949), species evenness (*J′*) (Pielou 1966), and species richness (*d*) (Margalef 1968) were calculated using the following formula:

$$H^{\prime }=-\sum_{i=1}^{s}\mathrm{Pi}\left(\ln\mathrm{Pi}\right)$$

$$J^{\prime }=\frac{H^{\prime }}{\ln\left(S\right)}$$

$$d=\frac{\left(S-1\right)}{\ln\left(N\right)}$$

where *S* is the total number of species in a sample, *P*_*i*_ = *ni*/*N* is the proportion of individuals in the total sample belonging to the *i*th species, *N*_*i*_ is the number of individuals belonging to the *i*th species, and *N* is the total number of individuals in a sample.

### 2.5 Statistical Analysis

All data were compiled in Microsoft Excel and expressed as mean ± standard deviation. Phytoplankton abundance data were log(*x* + 1) transformed prior to multivariate analyses to minimize skewness and mitigate the disproportionate influence of highly abundant taxa. Bray–Curtis similarity matrices were calculated on square‐root (or log‐transformed, see Supporting Information) abundance data for cluster analysis, ANOSIM and SIMPER. One‐way ANOSIM tests (999 permutations) were used to assess differences in assemblage composition among seasons and stations; significance was evaluated at *α* = 0.05. SIMPER analyses (999 permutations) were used to estimate taxa contributions to average dissimilarities between groups. For univariate tests we assessed normality with the Ryan–Joiner test and homogeneity of variances; parametric one‐way ANOVA was used when assumptions were met; otherwise, Kruskal–Wallis tests were applied. Tukey's pairwise test was applied to ascertain the difference in abundance, diversity indices, and physicochemical parameters between stations, followed by ANOVA. CCA was performed to relate dominant taxa to measured environmental variables; the significance of canonical axes was tested by Monte Carlo permutation (999 permutations). All multivariate and univariate analyses were performed in PAST v.3.0 (Hammer and Harper 2001); additional checks and plotting were carried out in R (version 4.3.2).

## 3 Results

### 3.1 Phytoplankton Composition and Seasonal Variation

**FIGURE 2:**
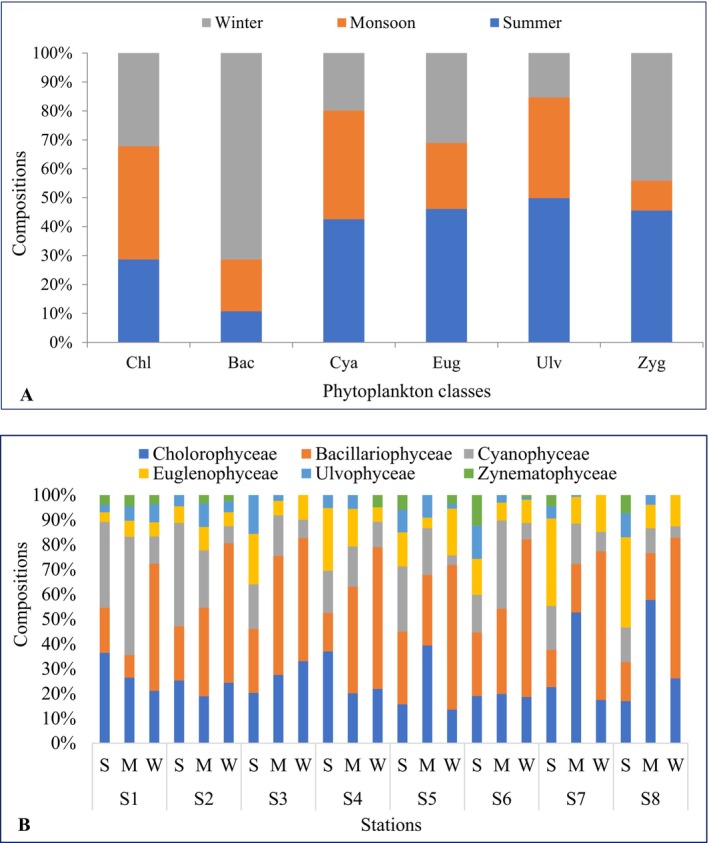
Composition (%) of phytoplankton classes in different (A) seasons and (B) stations in the Dakatia River, Chandpur, Bangladesh (Bac, Bacillariophyceae; Chl, Chlorophyceae; Cya, Cyanophyceae; Eug, Euglenophyceae; M, monsoon; S, summer; Ulv, Ulvophyceae; W, winter; Zyg, Zygnematophyceae).

A total of 37 phytoplankton genera, representing six major classes—Cyanophyceae (8 genera), Bacillariophyceae (10 genera), Chlorophyceae (12 genera), Euglenophyceae (3 genera), Ulvophyceae (1 genus), and Zygnematophyceae (3 genera)—were recorded from the Dakatia River, Chandpur, Bangladesh (Supporting Information S1). Of these, 13 genera were consistently present across all seasons, with 9 identified as dominant. Seasonal variation was evident, with 23 genera documented in summer (16 dominant), 30 genera in the monsoon (16 dominant), and 23 genera in winter (16 dominant). Phytoplankton diversity peaked during the monsoon (17 genera) and was lowest in summer (8 genera), reflecting the strong influence of seasonal hydrology on community composition. The mean composition of phytoplankton classes was Bacillariophyceae (36.50%), Chlorophyceae (25.91%), Cyanophyceae (17.00%), Euglenophyceae (13.20%), Ulvophyceae (5.06%), and Zygnematophyceae (2.31%). Seasonal and spatial variations in class composition are illustrated in Figure 2. Bacillariophyceae exhibited the highest occurrence (71.31%) in winter, whereas Zygnematophyceae was least abundant (10.35%) during monsoon (Figure 2A). The relative abundance of Chlorophyceae ranged from 13.51% (S_5_, winter) to 57.78% (S_8_, monsoon); Bacillariophyceae from 9.03% (S_1_, monsoon) to 63.55% (S_6_, winter); Cyanophyceae from 3.86% (S_5_, winter) to 47.74% (S_1_, monsoon); Euglenophyceae from 3.94% (S_1_, summer) to 36.36% (S_8_, summer); Ulvophyceae from 2.96% (S_1_, summer) to 15.63% (S_3_, summer); and Zygnematophyceae from 2.50% (S_2_, winter) to 12.29% (S_6_, summer) (Figure 2B).

### 3.2 Phytoplankton Abundance and Diversity

**FIGURE 3:**
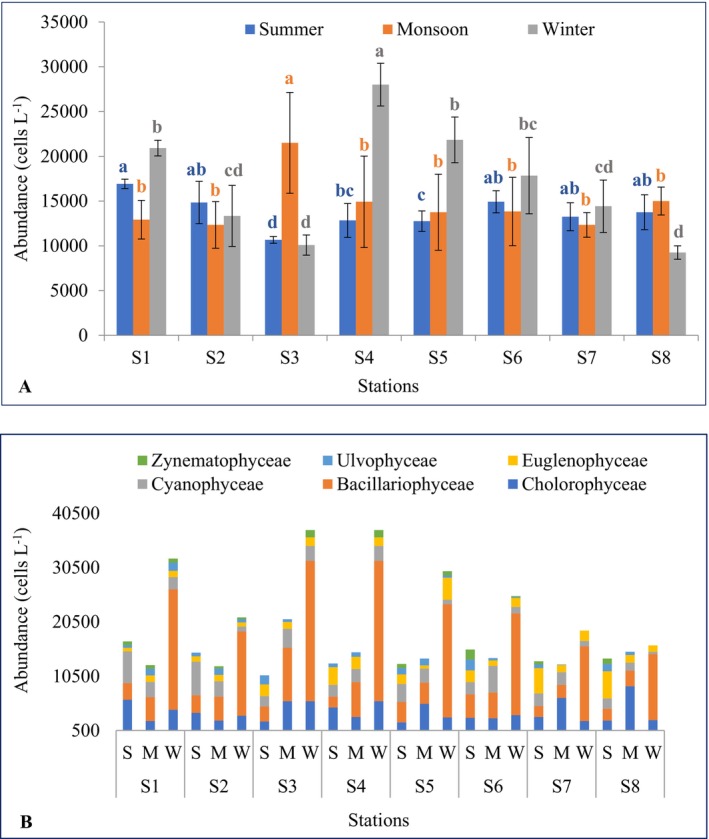
Abundance (cells L^−1^) of (A) total phytoplankton and (B) classes of phytoplankton in the Dakatia River, Chandpur, Bangladesh (M, monsoon; S, summer, and W, winter). Data are presented as mean ± standard deviation (*n* = 3). Means with different superscript (blue = summer, orange = monsoon, and gray = winter) are significantly different at *p* < 0.05.

Phytoplankton abundance showed marked spatial and seasonal variability (Figure 3A). In summer, abundance ranged from a maximum of 16,916.70 ± 520.42 cells L^−1^ at S_1_ to a minimum of 10,666.70 ± 381.88 cells L^−1^ at S_3_ (mean = 13,739.60 ± 1857.76 cells L^−1^). During the monsoon, the highest abundance was recorded at S_3_ (21,500.00 ± 5634.71 cells L^−1^) and the lowest at S_7_ (12,333.30 ± 1376.89 cells L^−1^), with a mean of 14,572.92 ± 2982.90 cells L^−1^. In winter, values peaked at S_4_ (28,000.00 ± 2384.85 cells L^−1^) and were lowest at S_8_ (9250.00 ± 750.00 cells L^−1^), yielding a mean of 16,958.33 ± 6418.75 cells L^−1^. Significant differences in abundance were observed across stations in summer (*F* = 4.44, *p* = 0.006) and winter (*F* = 18.59, *p* < 0.001), while differences among stations during the monsoon (*H* = 8.42, *p* = 0.29) and among seasons overall (*H* = 0.99, *p* = 0.61) were not significant. Maximum phytoplankton abundance in summer was found at S_1_, which was statistically identical (*p* > 0.05) to S_2_, S_6_, S_7_, and S_8_ but significantly higher (*p* < 0.05) than S_4_, S_5_, and S_3_ (Figure 3A). Significantly higher (*p* < 0.05) phytoplankton abundance was observed at S_3_ during monsoon and at S_4_ during winter than all other stations (Figure 3A).

Class‐level patterns further highlighted seasonal shifts in community structure (Figure 3B). Chlorophyceae abundance ranged from 6500.00 cells L^−1^ at S_7_ in the monsoon to 2000.00 cells L^−1^ at S_5_ in summer (mean = 3923.54 ± 1806.01 cells L^−1^). Bacillariophyceae varied widely, with maxima of 25,833.33 cells L^−1^ at S_3_ and S_4_ in winter and minima of 2000.00 cells L^−1^ at S_7_ in summer (mean = 9048.58 ± 8144.77 cells L^−1^). Cyanophyceae ranged from 6166.67 cells L^−1^ at S_2_ in summer to 416.67 cells L^−1^ at S_8_ in winter (mean = 2536.17 ± 1444.82 cells L^−1^). Euglenophyceae abundance ranged from 5000.00 cells L^−1^ at S_8_ in summer to 583.33 cells L^−1^ at S_5_ in the monsoon (mean = 1866.50 ± 1209.23 cells L^−1^). Ulvophyceae were less abundant, ranging from 2000.00 cells L^−1^ at S_6_ in summer to 83.33 cells L^−1^ at S_7_ in the monsoon (mean = 724.08 ± 573.47 cells L^−1^). Zygnematophyceae were consistently low, ranging from 1833.33 cells L^−1^ at S_6_ in summer to 333.33 cells L^−1^ at S_2_ in winter (mean = 441.50 ± 108.37 cells L^−1^).

**FIGURE 4:**
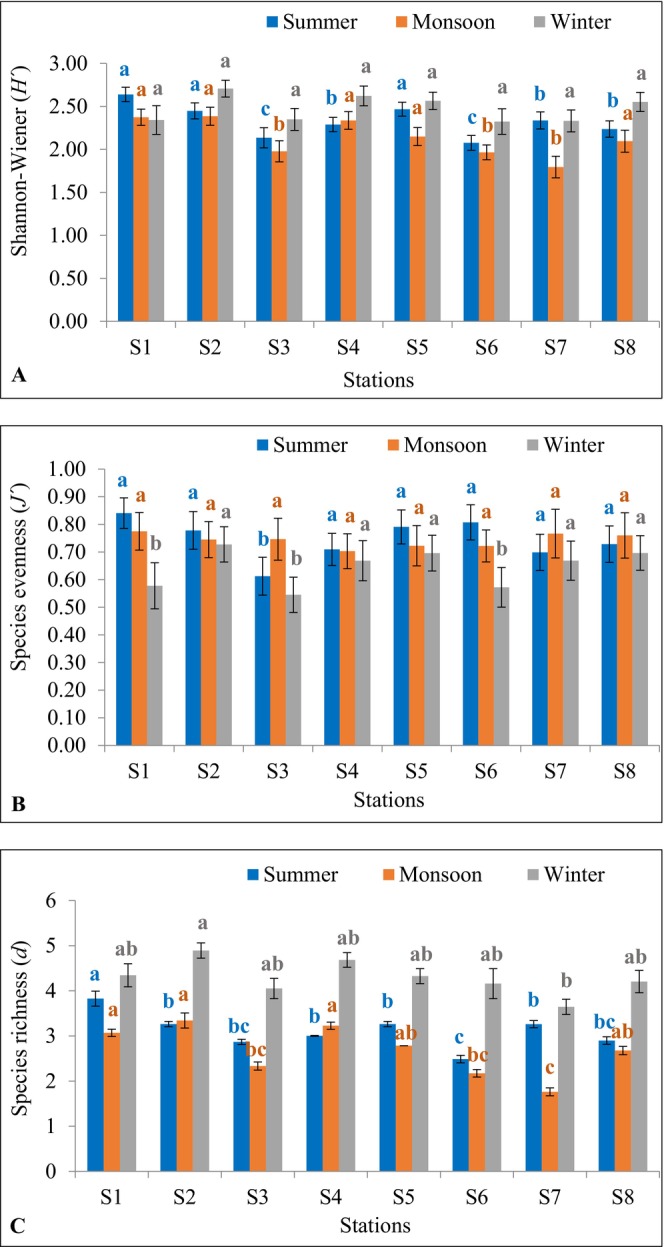
Diversity indices: (A) Shannon–Wiener, (B) Species evenness, and (C) Species richness of phytoplankton in the Dakatia River, Chandpur, Bangladesh (M, monsoon; S, summer; W, winter). Data are presented as mean ± standard deviation (*n* = 3). Means with different superscript (blue = summer, orange = monsoon, and gray = winter) are significantly different at *p* < 0.05.

Diversity indices, namely Shannon–Wiener diversity, species evenness, and species richness, are plotted in Figure 4. The highest Shannon–Wiener diversity was 2.64 ± 0.14 at S_1_ and the lowest was 2.08 ± 0.15 at S_6_ with a mean of 2.33 ± 0.19 during summer, the maximum was 2.39 ± 0.19 at S_2_ and the minimum was 1.79 ± 0.22 at S_7_ with a mean of 2.14 ± 0.22 in monsoon, and the higher was 2.71 ± 0.17 at S_2_ and the lower was 2.32 ± 0.26 at S_6_ with a mean of 2.47 ± 0.15 during winter (Figure 4A). Significant differences among stations were observed in Shannon–Wiener diversity during summer (*F* = 3.99, *p* = 0.01), monsoon (*F* = 3.95, *p* = 0.01), and among seasons (*F* = 6.56, *p* = 0.01), while no significant difference among stations was observed in winter (*F* = 1.49, *p* = 0.24). Maximum Shannon–Wiener diversity in summer was found at S_1_ which was statistically identical (*p* > 0.05) to S_2_ and S_5_ but significantly higher (*p* < 0.05) than S_7_, S_4_, S_8_, S_3_, and S_6_ (Figure 4A). Maximum Shannon–Wiener diversity during monsoon was observed at S_2_ which was statistically identical (*p* > 0.05) to S_1_, S_4_, S_5_, and S_8_ but significantly higher (*p* < 0.05) than S_3_, S_6_, and S_7_ (Figure 4A). Higher Shannon–Wiener diversity in winter was found at S_2_ which was statistically identical (*p* > 0.05) to all other stations (Figure 4A).

Maximum species evenness was 0.84 ± 0.10 at S_1_ and minimum was 0.61 ± 0.12 at S_3_ with a mean of 0.75 ± 0.07 in summer, highest was 0.77 ± 0.12 at S_1_ and lowest was 0.70 ± 0.11 at S_4_ with a mean of 0.74 ± 0.03 during monsoon, and higher was 0.73 ± 0.11 at S_2_ and lower was 0.55 ± 0.11 at S_3_ with a mean of 0.65 ± 0.07 in winter (Figure 4B). No significant difference was found in species evenness among stations during summer (*F* = 1.31, *p* = 0.31), monsoon (*F* = 0.12, *p* = 0.99), and winter (*F* = 0.98, *p* = 0.48). However, a significant difference was observed in species evenness among seasons (*F* = 7.49, *p* = 0.004). The highest species evenness during summer was found at S_1_ which was statistically identical (*p* > 0.05) to all other stations, except S_3_ (Figure 4B). Higher species evenness in monsoon was found at S_1_ which was statistically identical (*p* > 0.05) to all other stations (Figure 4B). Maximum species evenness during winter was found at S_2_ which was statistically identical (*p* > 0.05) to S_8_, S_5_, S_7_ and S_4_ but significantly higher (*p* < 0.05) than S_1_, S_6_, and S_3_ (Figure 4B).

The highest species richness was 3.83 ± 0.29 at S_1_ and the lowest was 2.15 ± 0.14 at S_6_ with a mean of 3.07 ± 0.48 in summer, maximum was 3.34 ± 0.29 at S_2_ and minimum was 1.76 ± 0.15 at S_7_ with a mean of 2.67 ± 0.55 during monsoon, and higher was 4.89 ± 0.29 at S_2_ and lower was 3.65 ± 0.29 at S_7_ with a mean of 4.29 ± 0.38 in winter (Figure 4C). Significant differences were found in species richness among stations during summer (*F* = 38.30, *p* = 7.91E‐09), monsoon (*F* = 33.36, *p* = 2.06E‐08), winter (*F* = 2.92, *p* = 0.04), and among seasons (*F* = 25.20, *p* = 2.63E‐06). Significantly higher (*p* < 0.05) species richness in summer was observed at S_1_ (Figure 4C). Maximum species richness during monsoon was found at S_2_, which was statistically identical (*p* > 0.05) to S_4_, S_1_, S_5_, and S_8_ but significantly higher (*p* < 0.05) than S_3_, S_6_, and S_7_ (Figure 4C). The highest species richness in winter was observed at S_2_, which was statistically identical (*p* > 0.05) to all other stations, except S_7_ (Figure 4C).

### 3.3 Phytoplankton Assemblage

**FIGURE 5:**
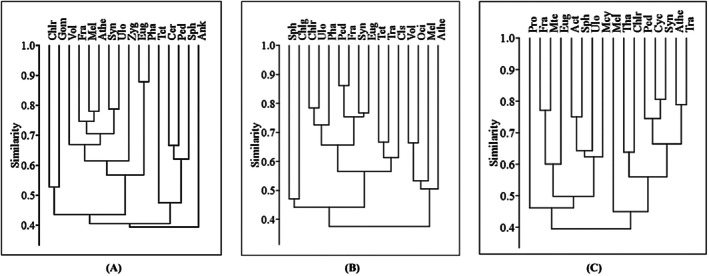
Dendrograms showing clusters based on the Bray–Curtis similarity matrix of 16 dominant genera during (A) summer, (B) monsoon, and (C) winter (Act, *Actinastrum* sp.; Ank, *Ankistrodesmus* sp.; Athe, *Aphanothece* sp.; Cer, *Ceratium* sp.; Chlg, *Chlorogonium* sp.; Chlr, *Chlorella* sp.; Cls, *Closterium* sp.; Cym, *Cymbella* sp.; Eug, *Euglena* sp.; Fra, *Fragilaria* sp.; Gom, *Gomphosphaeria* sp.; Mcy, *Microcystis* sp.; Mel, *Melosira* sp.; Mte, *Micrasterias* sp.; Oci, *Oscillatoria* sp.; Ped, *Pediastrum* sp.; Pha, *Phacus* sp.; Pro, *Prorocentrum* sp.; Sph, *Sphaerocystis* sp.; Syn, *Synedra* sp.; Tet, *Tetraedron* sp.; Tha, *Thalassiothrix* sp.; Tra, *Trachelomonas* sp.; Ulo, *Ulothrix* sp.; Vol, *Volvox* sp.; Zyg, *Zygnema* sp.).

Cluster analysis revealed no pronounced differentiation among phytoplankton genera, with five major clusters identified at a 50% similarity threshold across the summer, monsoon, and winter seasons (Figure 5). During summer, *Ankistrodesmus* sp. and *Tetraedron* sp. remained isolated; one cluster contained *Chlorella* sp. and *Gomphosphaeria* sp.; another contained *Ceratium* sp., *Pediastrum* sp., and *Sphaerocystis* sp.; while the largest cluster grouped the remaining nine genera (Figure 5A). In the monsoon, *Aphanothece* sp., *Chlorogonium* sp., and *Sphaerocystis* sp. remained isolated; one cluster contained *Melosira* sp., *Oscillatoria* sp., and *Volvox* sp.; and the largest cluster grouped the remaining 10 genera (Figure 5B). In winter, *Melosira* sp. and *Prorocentrum* sp. remained isolated; one cluster contained *Euglena* sp., *Fragilaria* sp., and *Micrasterias* sp.; another contained *Actinastrum* sp., *Microcystis* sp., *Sphaerocystis* sp., and *Ulothrix* sp.; while the largest cluster grouped the remaining seven genera (Figure 5C).

The analysis of similarity (ANOSIM) illustrated significant dissimilarity of phytoplankton among seasons (Global *R* = 0.877, *p* = 0.0001), while there was no dissimilarity among different stations (Global *R* = 0.027, *p* = 0.198). Additionally, significant dissimilarity (*p* < 0.001) prevailed in pair combinations of seasons, while no significant (*p* > 0.05) difference was observed in pair combinations of stations, except S_1_ with S_7_ and S_8_ (Table 1). According to the similarity percentage (SIMPER) analysis, phytoplankton assemblage was not found to be diverse between stations, and the genera *Melosira* sp. contributed mostly to the dissimilarity from station to station, followed by *Oscillatoria* sp. and *Gomphosphaeria* sp., respectively (Table 1).

**TABLE 1:** Outcomes of one‐way ANOSIM and SIMPER analysis of the phytoplankton abundance between different stations and seasons.

Groups	ANOSIM		SIMPER		
	R	P	Most discriminating genus (sp.)	SIMPER average dissimilarity	Contribution (%)
S_1_ versus S_2_	0.066	0.172	*Melosira*	9.48	16.15
S_1_ versus S_3_	0.085	0.140	*Melosira*	10.15	17.15
S1 versus S4	0.070	0.170	*Melosira*	9.47	14.72
S1 versus S5	0.148	0.070	*Melosira*	9.35	14.65
S1 versus S6	0.111	0.115	*Melosira*	9.43	14.78
S1 versus S7	0.234	0.035	*Oscillatoria*	12.08	16.79
S1 versus S8	0.198	0.040	*Melosira*	8.35	12.03
S2 versus S3	−0.069	0.786	*Melosira*	9.33	2.65
S2 versus S4	0.041	0.221	*Melosira*	8.40	13.77
S2 versus S5	−0.024	0.505	*Gomphosphaeria*	6.68	11.94
S2 versus S6	0.062	0.190	*Melosira*	6.30	10.50
S2 versus S7	0.052	0.211	*Oscillatoria*	12.86	19.47
S2 versus S8	0.059	0.198	*Gomphosphaeria*	6.35	9.91
S3 versus S4	−0.059	0.728	*Melosira*	10.49	17.12
S3 versus S5	−0.134	0.994	*Melosira*	8.44	14.58
S3 versus S6	0.048	0.223	*Melosira*	9.45	15.60
S3 versus S7	−0.024	0.510	*Oscillatoria*	13.64	21.07
S3 versus S8	0.022	0.302	*Melosira*	8.44	13.77
S4 versus S5	−0.025	0.504	*Melosira*	7.72	12.70
S4 versus S6	0.042	0.236	*Melosira*	7.78	12.40
S4 versus S7	0.054	0.211	*Oscillatoria*	12.75	19.01
S4 versus S8	0.024	0.284	*Melosira*	9.27	14.12
S5 versus S6	−0.043	0.626	*Melosira*	5.83	10.17
S5 versus S7	−0.053	0.683	*Oscillatoria*	13.19	20.49
S5 versus S8	−0.141	0.994	*Melosira*	5.91	9.80
S6 versus S7	0.007	0.328	*Oscillatoria*	12.17	18.76
S6 versus S8	0.055	0.224	*Melosira*	7.25	11.53
S7 versus S8	−0.039	0.598	*Oscillatoria*	12.51	19.61
Sum versus Mon	0.787	0.000	*Melosira*	8.88	14.41
Sum versus Win	1.000	0.000	*Melosira*	11.27	15.84
Mon versus Win	0.806	0.000	*Melosira*	6.14	10.50

### 3.4 Physicochemical Parameters

**FIGURE 6:**
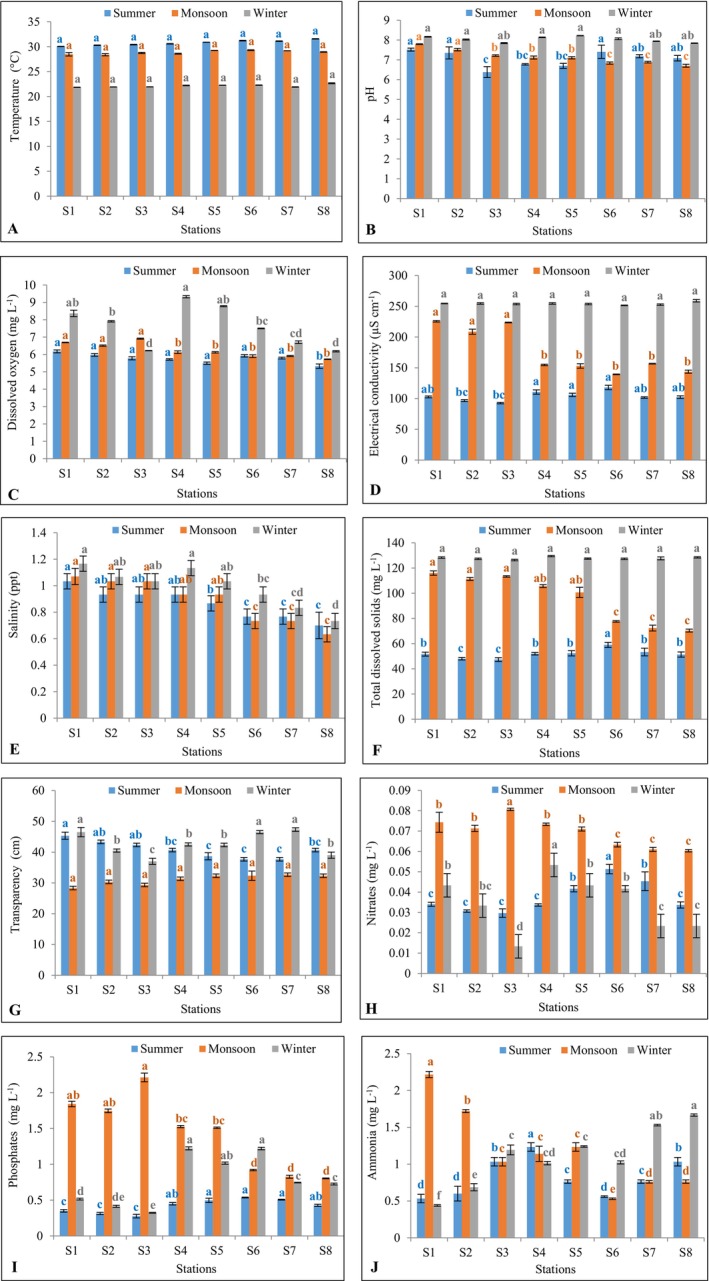
Mean physicochemical parameters at different stations of the Dakatia River, Chandpur, Bangladesh. Data are presented as mean ± standard deviation (*n* = 3). Means with different superscript (blue = summer, orange = monsoon, and gray = winter) are significantly different at *p* < 0.05. The subparts of the figure indicate the following water quality parameters: (A) Temperature, (B) pH, (C) Dissolved Oxygen, (D) Electrical Conductivity, (E) Salinity, (F) Total Dissolved Solids, (G) Transparency, (H) Nitrates, (I) Phosphates, and (J) Ammonia.

The physicochemical characteristics of the Dakatia River are presented in Figure 6. Water temperature exhibited marked seasonal variation, with the highest values recorded during summer (30.05°C ± 0.03°C at S1 to 31.58°C ± 0.05°C at S8; mean = 30.77°C ± 0.52°C), moderate values during the monsoon (28.48°C ± 0.35°C at S1 to 29.30°C ± 0.10°C at S5; mean = 28.86°C ± 0.36°C), and the lowest values during winter (21.85°C ± 0.03°C at S1 to 22.66°C ± 0.10°C at S8; mean = 22.13°C ± 0.27°C). These differences were statistically significant across stations within each season (summer: *F* = 301.80, *p* < 0.001; monsoon: *F* = 15.36, *p* < 0.001; winter: *F* = 95.24, *p* < 0.001) and among seasons overall (*H* = 20.48, *p* < 0.001). While no significant difference (*p* > 0.05) in temperature was identified between stations during summer, monsoon, and winter (Figure 6A).

pH values also varied significantly, ranging from slightly acidic to alkaline. In summer, values ranged from 6.38 ± 0.27 at S3 to 7.51 ± 0.07 at S1 (mean = 7.05 ± 0.40). During the monsoon, pH ranged from 6.70 ± 0.08 at S8 to 7.79 ± 0.02 at S1 (mean = 7.15 ± 0.36), while in winter it was consistently alkaline, from 7.85 ± 0.03 at S3 to 8.22 ± 0.01 at S5 (mean = 8.03 ± 0.15). Significant differences were observed both across stations (summer: *F* = 11.50, *p* < 0.001; monsoon: *F* = 126.50, *p* < 0.001; winter: *F* = 123.50, *p* < 0.001) and among seasons (*F* = 22.61, *p* < 0.001). Maximum pH in summer was found at S_1_, which was statistically identical (*p* > 0.05) to S_6_, S_2_, S_7_, and S_8_ but significantly higher (*p* < 0.05) than S_4_, S_5_, and S_3_ (Figure 6B). Significantly higher pH during monsoon was observed at S_1_ and S_2_ than all other stations (Figure 6B). Maximum pH in winter was found at S_1_, which was statistically identical (*p* > 0.05) to all other stations (Figure 6B).

Dissolved oxygen (DO) showed pronounced seasonal variation. In summer, DO ranged from 5.33 ± 0.13 mg L^−1^ at S8 to 6.18 ± 0.08 mg L^−1^ at S1 (mean = 5.77 ± 0.27 mg L^−1^). During the monsoon, values ranged from 5.72 ± 0.02 mg L^−1^ at S8 to 6.91 ± 0.03 mg L^−1^ at S3 (mean = 6.24 ± 0.42 mg L^−1^). In winter, concentrations increased substantially, ranging from 6.19 ± 0.04 mg L^−1^ at S8 to 9.33 ± 0.07 mg L^−1^ at S4 (mean = 7.62 ± 1.18 mg L^−1^). Differences were significant across stations and seasons (summer: *F* = 34.86, *p* < 0.001; monsoon: *F* = 246.0, *p* < 0.001; winter: *F* = 693.0, *p* < 0.001; seasonal: *H* = 14.71, *p* = 0.001). The highest dissolved oxygen during summer was found at S_1_, which was statistically identical (*p* > 0.05) to all other stations, except S_8_ (Figure 6C). Maximum dissolved oxygen in monsoon was observed at S_3_, which was statistically identical (*p* > 0.05) to S_1_ and S_2_ but significantly higher (*p* < 0.05) than all other stations (Figure 6C). Maximum dissolved oxygen during winter was found at S_4_, which was statistically identical (*p* > 0.05) to S_5_ and S_1_ but significantly higher (*p* < 0.05) than all other stations (Figure 6C).

Electrical conductivity (EC) increased progressively from summer to winter. In summer, EC ranged from 92.67 ± 1.15 μS cm^−1^ at S3 to 118.0 ± 3.61 μS cm^−1^ at S6 (mean = 103.83 ± 7.90 μS cm^−1^). During the monsoon, values were higher, ranging from 139.33 ± 0.58 μS cm^−1^ at S6 to 225.67 ± 1.15 μS cm^−1^ at S1 (mean = 175.67 ± 36.94 μS cm^−1^). In winter, EC reached its peak, varying from 251.50 ± 0.50 μS cm^−1^ at S7 to 259.0 ± 2.0 μS cm^−1^ at S8 (mean = 254.29 ± 2.19 μS cm^−1^). These seasonal and spatial variations were statistically significant (summer: *F* = 34.01, *p* < 0.001; monsoon: *F* = 266.20, *p* < 0.001; winter: *F* = 10.35, *p* < 0.001; seasonal: *H* = 20.48, *p* < 0.001). Maximum electrical conductivity during summer was found at S_6_, which was statistically identical (*p* > 0.05) to S_4_ and S_5_ but significantly higher (*p* < 0.05) than all other stations (Figure 6D). Maximum electrical conductivity in monsoon was observed at S_1_, which was statistically identical (*p* > 0.05) to S_3_ and S_2_ but significantly higher (*p* < 0.05) than all other stations (Figure 6D). The highest electrical conductivity during winter was found at S_8_, which was statistically identical (*p* > 0.05) to all other stations (Figure 6D).

Salinity ranged from 0.70 ± 0.10 ppt at S_8_ to 1.03 ± 0.06 ppt at S_1_ during summer (mean = 0.86 ± 0.10 ppt), 0.63 ± 0.06 ppt at S_8_ to 1.07 ± 0.06 ppt at S_1_ during monsoon (mean = 0.88 ± 0.16 ppt), and 0.73 ± 0.06 ppt at S_8_ to 1.17 ± 0.06 ppt at S_1_ during winter (mean = 0.99 ± 0.15 ppt). Significant differences in salinity were found across stations during summer (*F* = 8.70, *p* = 0.000), monsoon (*F* = 23.14, *p* = 0.000), and winter (*F* = 19.93, *p* = 0.000), while no significant difference was noted among seasons (*F* = 2.01, *p* = 0.16). Maximum salinity during summer, monsoon, and winter was found at S_1_, which was statistically identical (*p* > 0.05) to S_2_, S_3_, S_4_, and S_5_ but significantly higher (*p* < 0.05) than S_6_, S_7_, and S_8_ (Figure 6E).

Total dissolved solids ranged from 47.33 ± 1.53 mg L^−1^ at S_3_ to 59.0 ± 2.0 mg L^−1^ at S_6_ during summer (mean = 51.88 ± 3.57 mg L^−1^), 70.33 ± 1.15 mg L^−1^ at S_8_ to 116.0 ± 1.73 mg L^−1^ at S_1_ during monsoon (mean = 95.92 ± 9.29 mg L^−1^), and 126.33 ± 0.58 mg L^−1^ at S_3_ to 129.5 ± 0.5 mg L^−1^ at S_4_ during winter (mean = 127.81 ± 9.5 mg L^−1^). Significant differences in total dissolved solids were found across stations during summer (*F* = 10.66, *p* = 0.000), monsoon (*F* = 446.10, *p* = 0.000), and winter (*F* = 6.38, *p* = 0.001), as well as among seasons (*H* = 20.48, *p* = 0.000). Significantly higher (*p* < 0.05) total dissolved solids were found at S_6_ than all other stations during summer (Figure 6F). Maximum total dissolved solids in monsoon were observed at S_1_ which was statistically identical (*p* > 0.05) to S_3_, S_2_, S_4_ and S_5_ but significantly higher (*p* < 0.05) than S_6_, S_7_, and S_8_ (Figure 6F). The highest total dissolved solids in winter were found at S_4_ which was statistically identical (*p* > 0.05) to all other stations (Figure 6F).

Water transparency ranged from 37.67 ± 0.58 cm at S_6_ to 45.33 ± 1.15 cm at S_1_ during summer (mean = 40.79 ± 2.77 cm), 28.33 ± 0.58 cm at S_1_ to 32.67 ± 0.58 cm at S_7_ during monsoon (mean = 31.13 ± 1.62 cm), and 37.00 ± 1.00 cm at S_3_ to 47.33 ± 0.58 cm at S_7_ during winter (mean = 42.71 ± 3.81 cm). Significant differences in water transparency were found across stations during summer (*F* = 39.34, *p* = 0.000), monsoon (*F* = 13.54, *p* = 0.000), and winter (*F* = 61.44, *p* = 0.000), as well as among seasons (*F* = 37.32, *p* = 0.000). Maximum transparency in summer was observed at S_1_, which was statistically identical (*p* > 0.05) to S_2_ and S_3_ but significantly higher (*p* < 0.05) than all other stations (Figure 6G). The highest transparency during monsoon was found at S_7_, which was statistically identical (*p* > 0.05) to all other stations (Figure 6G). Maximum transparency in winter was observed at S_7_, which was statistically identical (*p* > 0.05) to S_1_ and S_6_ but significantly higher (*p* < 0.05) than all other stations (Figure 6G).

Nitrate concentrations ranged from 0.03 ± 0.002 mg L^−1^ at S_3_ to 0.05 ± 0.002 mg L^−1^at S_6_ during summer (mean = 0.04 ± 0.01 mg L^−1^), 0.06 ± 0.001 mg L^−1^ at S_8_ to 0.08 ± 0.001 mg L^−1^ at S_3_ during monsoon (mean = 0.07 ± 0.01 mg L^−1^), and 0.01 ± 0.006 mg L^−1^ at S_3_ to 0.05 ± 0.01 mg L^−1^ at S_4_ during winter (mean = 0.03 ± 0.01 mg L^−1^). Significant differences in nitrate concentrations were observed across stations during summer (*F* = 38.55, *p* = 0.000), monsoon (*F* = 40.10, *p* = 0.000), and winter (*F* = 18.34, *p* = 0.000), as well as among seasons (*F* = 30.92, *p* = 0.000). Significantly higher (*p* < 0.05) nitrate was found at S_6_ during summer, at S_3_ during monsoon, and at S_4_ during winter than at all other stations (Figure 6H).

Phosphate concentrations ranged from 0.28 ± 0.03 mg L^−1^ at S_3_ to 0.54 ± 0.01 mg L^−1^ at S_6_ during summer (mean = 0.42 ± 0.10 mg L^−1^), 0.80 ± 0.01 mg L^−1^at S_8_ to 2.14 ± 0.14 mg L^−1^ at S_3_ during monsoon (mean = 1.42 ± 0.52 mg L^−1^), and 0.33 ± 0.01 mg L^−1^ at S_3_ to 1.22 ± 0.02 mg L^−1^ at S_4_ during winter (mean = 0.77 ± 0.35 mg L^−1^). Significant differences in phosphate concentrations were detected across stations during summer (*F* = 4.12, *p* = 0.01), monsoon (*F* = 290.20, *p* = 0.000), and winter (*F* = 1903.0, *p* = 0.000), as well as among seasons (*H* = 14.86, *p* = 0.000). Maximum phosphate during summer was observed at S_6_, which was statistically identical (*p* > 0.05) to S_7_, S_5_, S_4_, and S_8_ but significantly higher (*p* < 0.05) than S_1_, S_2_, and S_3_ (Figure 6I). Significantly higher (*p* < 0.05) phosphate was found at S_3_ in monsoon than at all other stations (Figure 6I). Significantly higher (*p* < 0.05) phosphate was observed at S_4_ and S_6_ during winter than at all other stations (Figure 6I).

Ammonia concentrations exhibited spatial variability within seasons but did not differ significantly across seasons overall. During summer, concentrations ranged from 0.53 ± 0.06 mg L^−1^ at S1 to 1.23 ± 0.06 mg L^−1^ at S4 (mean = 0.81 ± 0.27 mg L^−1^). In the monsoon, values were broader, ranging from 0.53 ± 0.01 mg L^−1^ at S6 to 2.22 ± 0.04 mg L^−1^ at S1 (mean = 1.17 ± 0.56 mg L^−1^). Winter concentrations varied from 0.44 ± 0.01 mg L^−1^ at S1 to 1.67 ± 0.02 mg L^−1^ at S8 (mean = 1.10 ± 0.41 mg L^−1^). Significant spatial differences were observed across stations in all three seasons (summer: *F* = 95.90, *p* < 0.001; monsoon: *F* = 216.60, *p* < 0.001; winter: *F* = 22.04, *p* < 0.001), whereas seasonal differences were not significant (*F* = 1.61, *p* = 0.22). Significantly higher (*p* < 0.05) ammonia was found at S_4_ during summer, at S_1_ during monsoon, and at S_8_ during winter (except with S_7_) than at all other stations (Figure 6J).

### 3.5 Relationship Between Ecological and Biological Variables

The correlation analysis revealed several notable relationships between phytoplankton groups, community indices, and environmental variables (Supporting Information S2). During summer, members of Euglenophyceae exhibited a significant positive correlation with temperature (*r* = 0.77, *p* < 0.05) (Supporting Information S2A). Chlorophyceae showed significant positive associations with dissolved oxygen (*r* = 0.68, *p* < 0.05) and salinity (*r* = 0.87, *p* < 0.01), while Zygnematophyceae were positively correlated with total dissolved solids (*r* = 0.86, *p* < 0.01), phosphate (*r* = 0.81, *p* < 0.05), and nitrate (*r* = 0.79, *p* < 0.05) (Supporting Information S2A). In summer, total phytoplankton abundance showed a significant negative correlation with ammonia (*r* = −0.74, *p* < 0.05). Salinity, in contrast, was positively correlated with both Shannon–Wiener diversity (*r* = 0.81, *p* < 0.05) and species richness (*r* = 0.74, *p* < 0.05) (Supporting Information S2A). During the monsoon, Bacillariophyceae were positively associated with phosphate (*r* = 0.72, *p* < 0.05) and nitrate (*r* = 0.83, *p* < 0.05), while Zygnematophyceae showed strong positive correlations with pH (*r* = 0.88, *p* < 0.01) and ammonia (*r* = 0.91, *p* < 0.01), but a negative relationship with transparency (*r* = −0.74, *p* < 0.05). Ulvophyceae were also positively correlated with ammonia (*r* = 0.80, *p* < 0.05). At the community level, ammonia exhibited significant positive correlations with Shannon–Wiener diversity (*r* = 0.80, *p* < 0.05) and species richness (*r* = 0.72, *p* < 0.05), whereas temperature showed negative correlations with both diversity (*r* = −0.77, *p* < 0.05) and richness (*r* = −0.76, *p* < 0.05) (Supporting Information S2B). In winter, the only significant relationship detected was a negative correlation between Ulvophyceae and ammonia (*r* = −0.78, *p* < 0.05) (Supporting Information S2C).

**FIGURE 7:**
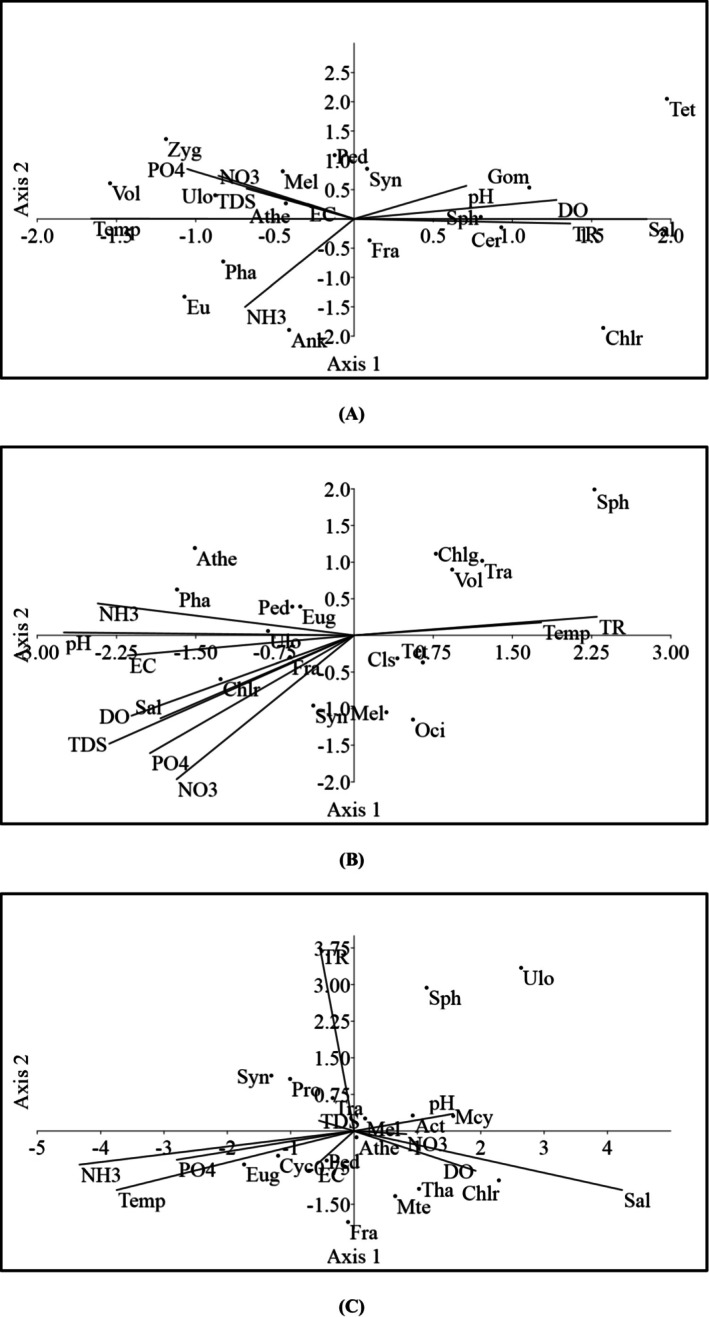
Canonical correspondence analysis (CCA) biplots between 16 dominant genera and physicochemical parameters during (A) summer, (B) monsoon, and (C) winter. (Act, *Actinastrum* sp.; Ank, *Ankistrodesmus* sp.; Athe, *Aphanothece* sp.; Cer, *Ceratium* sp.; Chlg, *Chlorogonium* sp.; Chlr, *Chlorella* sp.; Cls, *Closterium* sp.; Cym, *Cymbella* sp.; Eug, *Euglena* sp.; Fra, *Fragilaria* sp.; Gom, *Gomphosphaeria* sp.; Mcy, *Microcystis* sp.; Mel, *Melosira* sp.; Mte, *Micrasterias* sp.; Oci, *Oscillatoria* sp.; Ped, *Pediastrum* sp.; Pha, *Phacus* sp.; Pro, *Prorocentrum* sp.; Sph, *Sphaerocystis* sp.; Syn, *Synedra* sp.; Tet, *Tetraedron* sp.; Tha, *Thalassiothrix* sp.; Tra, *Trachelomonas* sp.; Ulo, *Ulothrix* sp.; Vol, *Volvox* sp.; Zyg, *Zygnema* sp.).

CCA has been drawn between 10 ecological parameters and 16 dominant phytoplankton genera for summer, monsoon, and winter that have been plotted in Figure 7. Eigenvalue of axis 1 (0.181) stated 41.21% correlation and axis 2 (0.135) stated 30.67% correlation between physicochemical parameters and the dominant genus of phytoplankton during summer. Temperature, salinity, and ammonia have maximum impact, while transparency, dissolved oxygen, and phosphate have moderate impact on the abundance of phytoplankton genera (Figure 7A). The abundance of *Gomphosphaeria* sp., *Tetraedron* sp., *Synedra* sp., *Volvox* sp., *Phacus* sp., *Fragilaria* sp., and *Euglena* sp. was highly correlated with environmental variables. The abundance of *Gomphosphaeria* sp., *Tetraedron* sp., and *Synedra* sp. was highly correlated with both axes as well as pH and DO. *Zygnema* sp., *Volvox* sp., *Melosira* sp., and *Pediastrum* sp. were positively correlated with phosphate, nitrate, and total dissolved solids. *Phacus* sp., *Euglena* sp., and *Ankistrodesmus* sp. were negatively correlated with both axes and ammonia.

Eigenvalue of axis 1 (0.168) narrated 44.12% correlation, and axis 2 (0.121) narrated 31.76% correlation between physicochemical parameters and the dominant genus of phytoplankton during monsoon. Nitrate, total dissolved solids, and phosphate indicated the highest relationship, while transparency, pH, dissolved oxygen, and ammonia indicated a higher relationship in the abundance of dominant phytoplankton genera (Figure 7B). The abundance of *Chlorella* sp., *Fragilaria* sp., *Chlorogonium* sp., *Volvox* sp., *Euglena* sp., and *Synedra* sp. was highly correlated with environmental variables. *Chlorella* sp., *Fragilaria* sp., and *Synedra* sp. were negatively correlated with both axes. *Phacus* sp., *Pediastrum* sp., *Ulothrix* sp., and *Euglena* sp. indicated positive relationships with ammonia.

Eigenvalue of axis 1 (0.137) stated 59.3% correlation and axis 2 (0.041) stated 17.38% correlation between physicochemical parameters and the dominant genus of phytoplankton during winter. Ammonia, temperature, salinity, and transparency have maximum impact on the abundance of dominant phytoplankton genera (Figure 7C). The abundance of *Euglena* sp., *Fragilaria* sp., *Pediastrum* sp., *Aphanothece* sp., *Microcystis* sp., *Chlorogonium* sp., and *Actinastrum* sp. was highly correlated with environmental variables. *Aphanothece* sp., *Microcystis* sp., *Chlorella* sp., and *Thalassiothrix* sp. were highly correlated with salinity and dissolved oxygen. *Euglena* sp., *Fragilaria* sp., *Pediastrum* sp., and *Cycotella* sp. were negatively correlated with both axes.

Overall, based on the above analyses (abundance data, correlation and CCA), changes in seasonal fluctuations in phytoplankton number and composition were closely linked to environmental circumstances. With the help of increasing nitrate and phosphate, diatoms often predominated in the winter, but during the monsoon, when nutrient‐rich discharge raised ammonia concentrations, green algae and euglenoids significantly increased. Because cyanobacteria can withstand greater temperatures and less nutritional availability, they were more common in the summer. Dinoflagellates were erratic and had little connection to their surroundings. Table 2 provides a summary of these trends, highlighting the major groups' seasonal peaks and the primary environmental factors that influence them.

**TABLE 2:** An overview of the main phytoplankton groups found in the Dakatia River, along with information on their seasonal abundance trends and related environmental factors.

Phytoplankton taxa	Key taxa	Seasonal abundance peak	Major environmental drivers
Bacillariophyceae	*Fragilaria*, *Navicula*, *Cyclotella*	Winter, moderate in monsoon	Positively correlated with nitrate and phosphate; negatively correlated with ammonia in summer
Chlorophyceae	*Pediastrum*, *Ulothrix*, *Scenedesmus*	Monsoon, early winter	Positively correlated with ammonia and phosphate in monsoon; supported by nutrient enrichment from runoff
Cyanophyceae	*Oscillatoria*, *Anabaena*	Summer, late monsoon	Correlated with higher temperature and lower flow; tolerant of reduced nutrient availability
Euglenophyceae	*Euglena*, *Phacus*	Monsoon and winter	Strongly favored by high NH_3_ and organic load during runoff periods
Dinophyceae	*Peridinium*	Low abundance, sporadic	Weak associations; occasionally correlated with higher salinity and warmer conditions

## 4 Discussion

### 4.1 Phytoplankton Community

A total of 37 phytoplankton genera representing six taxonomic classes were identified, with Bacillariophyceae and Chlorophyceae as the dominant groups. This composition is comparable to those reported from other major riverine and estuarine systems, including the Meghna River (41 genera, 7 classes; Hossain et al. 2016), the Halda River (34 genera, 5 classes; Hossain, Sarker, et al. 2022), the Pasur River Estuary (38 genera, 10 classes; Hasan et al. 2022), the Kohelia Channel of the northern Bay of Bengal (42 genera, 8 classes; Mamun et al. 2019), the Teesta River in India (35 genera, 7 classes; Das et al. 2022), and the Kailash Khal wetland in the Indian Sundarbans (36 genera, 8 classes; Gogoi et al. 2019). The community was dominated by Bacillariophyceae (36.50%) and Chlorophyceae (25.91%), a dominance pattern consistent with observations from other major riverine ecosystems of Bangladesh, including the Meghna River Estuary (Shaha et al. 2022), the Pasur River Estuary (Hasan et al. 2022), and the Padma River (Haque et al. 2019). Distinct spatiotemporal variations in community composition were observed, likely reflecting fluctuations in physicochemical conditions that influence phytoplankton structure and diversity (Gogoi et al. 2019; Mamun et al. 2019; Iqbal et al. 2017).

Seasonal dynamics revealed the highest phytoplankton diversity during the monsoon (30 genera), followed by summer (23 genera) and winter (22 genera). This seasonal disparity can be attributed to increased nitrate and phosphate availability during the monsoon, likely resulting from terrestrial runoff and hydrological connectivity, coupled with optimal physicochemical conditions favoring phytoplankton proliferation. These findings highlight the critical role of nutrient dynamics in regulating primary production and community structuring in subtropical riverine ecosystems.

The genera *Aphanothece* sp. (Cyanophyceae), *Fragilaria* sp., *Melosira* sp., and *Synedra* sp. (Bacillariophyceae), *Chlorella* sp., *Pediastrum* sp., and *Sphaerocystis* sp. (Chlorophyceae), *Euglena* sp. (Euglenophyceae), and *Ulothrix* sp. (Ulvophyceae) were consistently dominant across all seasons in the Dakatia River, Chandpur, Bangladesh. These taxa have also been reported as dominant in the Meghna River (Hossain et al. 2016), Padma River (Haque et al. 2019), and Karatoya River (Haque et al. 2021), highlighting their ecological significance in major river systems. Notably, these phytoplankton taxa have been identified in the gut contents of *Tenualosa ilisha* (Hilsa Shad), a commercially important species in the Meghna River (Sarker, Hossain, et al. 2023; Sarker, Sarker, et al. 2023; Shaha et al. 2022; Hasan et al. 2016), as well as in Indian major carps (Khan and Siddiqui 1973; Dewan et al. 1991; Khabade 2015), which inhabit natural riverine and floodplain ecosystems in Bangladesh (DOF 2023). The prevalence of these phytoplankton species underscores the fisheries potential of the Dakatia River, as they serve as primary food sources for economically valuable fish species, reinforcing its ecological and economic importance.

### 4.2 Phytoplankton Abundance and Diversity

The phytoplankton abundance in the Dakatia River exhibited considerable seasonal variation, with the highest recorded density reaching 28,000 ± 2384.85 cells L^−1^ and the lowest at 9250 ± 750.00 cells L^−1^. Comparable phytoplankton densities were observed in the Karatoya River (9430–24,200 cells L^−1^; Haque et al. 2021), the Kohelia Channel of the northern Bay of Bengal (6524–27,736 cells L^−1^; Mamun et al. 2019), and the Reju Khal Estuary along the southeastern coast of Bangladesh (9408–21,964 cells L^−1^; Iqbal et al. 2017), highlighting the ecological productivity and fisheries potential of the Dakatia River.

Seasonal dynamics revealed peak phytoplankton abundance in winter (16,958.33 ± 6418.75 cells L^−1^), while the lowest mean density was observed in summer (13,739.60 ± 1857.76 cells L^−1^). The increased phytoplankton abundance in winter is likely driven by enhanced water transparency, facilitating optimal light penetration for photosynthesis, coupled with elevated pH, dissolved oxygen, and total dissolved solids. Conversely, the decline in summer abundance may be attributed to reduced water quality parameters and increased biological interactions. Similar seasonal trends have been observed in the Pasur River estuary (Hasan et al. 2022), the Karatoya River (Haque et al. 2021), the Kohelia channel (Mamun et al. 2019), and the Jamuna River (Akter et al. 2018) of Bangladesh. The observed spatial and seasonal variability in phytoplankton abundance underscores the critical role of physicochemical parameters in regulating phytoplankton dynamics (Gogoi et al. 2019; Mamun et al. 2019; Iqbal et al. 2017).

The Shannon–Wiener diversity index (1.79 to 2.71) indicated a moderate level of phytoplankton diversity in the Dakatia River, aligning with similar observations from the Pasur River estuary (Hasan et al. 2022), Kohelia channel of the northern Bay of Bengal (Mamun et al. 2019), and Kailash khal wetland in the Indian Sundarbans (Gogoi et al. 2019). Species evenness values (0.55 to 0.84) suggested a moderately stable phytoplankton community, comparable to those in the Pasur River estuary (Hasan et al. 2022), Kohelia channel of the northern Bay of Bengal (Mamun et al. 2019), and Padma River (Haque et al. 2019). Additionally, species richness (1.76 to 4.89) highlighted the stability of the phytoplankton community, consistent with findings from the Padma River (Haque et al. 2019), Kohelia channel of the northern Bay of Bengal (Mamun et al. 2019), and Kailash khal wetland (Gogoi et al. 2019). These diversity indices reinforce the notion that physicochemical parameters and seasonal hydrodynamics significantly shape phytoplankton community structure in subtropical river systems.

### 4.3 Phytoplankton Assemblage

In the present study, significant dissimilarities (*p* < 0.001) were detected in phytoplankton assemblages among seasons and their pairwise combinations, whereas no significant variation (*p* > 0.05) was observed among stations or their pairwise comparisons in the Dakatia River, Bangladesh. The pronounced seasonal differences likely reflect fluctuations in physicochemical conditions across seasons that influence community composition and structure. Insignificant differences among different stations and pair combinations of stations were attributed to low‐distance, similar human and fisheries activities in the studied stations of the Dakatia River. Hasan et al. (2022) reported significant seasonal differences (*p* < 0.05) between the dry and wet periods, but no significant spatial variation (*p* > 0.05) among sampling sites in the Pasur River Estuary, Bangladesh. Similarly, Mamun et al. (2019) observed significant (*p* < 0.05) seasonal variations in phytoplankton community structure among and between seasonal pairs in the Kohelia Channel of the northern Bay of Bengal, Bangladesh. For the phytoplankton assemblage, no variation in dominant genus was observed from station to station and season to season during this study. The most dominant genus for dissimilarity at different stations and seasons was *Melosaria* sp., along with *Oscillatoria* sp., and *Gomphosphaeria* sp. Haque et al. (2021) found *Melosira* spp. as the most dominant species responsible for dissimilarity in phytoplankton assemblage in the Karatoya River of Bangladesh. Cluster analysis generated five major clusters at a similarity of 50%, which stated no significant differentiation of phytoplankton genus. This phenomenon revealed an unwavering pattern of phytoplankton community in the Dakatia River of Bangladesh.

### 4.4 Physicochemical Parameters

The physicochemical parameters play an indispensable role in determining diversity and community succession and thus can favor or hinder the growth of various phytoplankton (Aktan et al. 2005). During this study, significant differences were observed in the studied physicochemical parameters among stations during summer, monsoon, and winter, as well as among seasons, except salinity and ammonia, which exhibited no significant difference among seasons. The values of physicochemical parameters in this study are coherent with other studies that were conducted in the Karatoya River of Bangladesh (Haque et al. 2021), Kohelia channel of the northern Bay of Bengal of Bangladesh (Mamun et al. 2019), Kailash khal wetland of the Indian Sundarbans (Gogoi et al. 2019), and Meghna River of Bangladesh (Hossain et al. 2016). Maximum values of the studied physicochemical parameters were observed in winter except temperature, nitrate, phosphate, and ammonia. The highest value of temperature in summer may be due to maximum day length coupled with the highest intensity of light than in monsoon and winter, which is coherent with Haque et al. (2021). The concentrations of nitrate, phosphate, and ammonia were highest during monsoon compared to winter and monsoon may be due to the surface runoff caused by heavy rainfall, which transports street waste and organic matter that can enhance the nutrients of aquatic bodies (Sarker et al. 2021).

### 4.5 Relationships Between Physicochemical and Biological Variables

The correlation analyses demonstrate a strong correlation between phytoplankton and water quality variations in the Dakatia River, with ammonia playing a particularly significant role among the variables tested. The fact that the relationship varied with seasons. For example, ammonia had a negative correlation with total phytoplankton and a number of categories, including diatoms, during the summer. This is probably due to the combined effects of higher pH, higher temperature, and low discharge, which increase oxidation and speed up microbial nitrification, converting ammonium into nitrate more quickly and decreasing its availability for algal uptake (Haque et al. 2021; Iqbal et al. 2017). The observed negative relationships likely reflect the disadvantage experienced by nutrient‐sensitive taxa, such as diatoms, under such conditions. Comparable patterns of summertime nutrient depletion have been reported in the coastal channels and the Karatoya River of Bangladesh, influencing phytoplankton community dynamics (Mamun et al. 2019; Gogoi et al. 2019).

On the other hand, ammonia showed a favorable correlation with a number of taxa, including euglenoids and green algae, during the monsoon. While cooler water temperatures and decreased transparency inhibited nitrification and ammonia loss, heavy rainfall and surface runoff from aquaculture and agricultural operations probably supplied significant ammonium inputs (Hasan et al. 2022; Sarker et al. 2021). Because there was more ammonium available, opportunistic species that could take advantage of transient nutrient pulses were favored. In the Pasur and Meghna River systems, similar nutrient‐driven increases in phytoplankton growth during the monsoon season have been reported (Hossain et al. 2016; Hasan et al. 2022).

Canonical correspondence analysis (CCA) further substantiated these findings by demonstrating a robust relationship between dominant phytoplankton genera and key physicochemical parameters. The taxa *Chlorogonium* sp., *Euglena* sp., *Fragilaria* sp., *Synedra* sp., and *Volvox* sp. exhibited distinct responses to variations in ammonia, temperature, salinity, nitrate, and phosphate, indicating their ecological preference for specific environmental conditions. Such species‐specific affinities align with previous studies that identified temperature, transparency, salinity, nitrate, and phosphate as primary determinants of phytoplankton community structure (Das et al. 2022; Gogoi et al. 2019; Haque et al. 2019; Mamun et al. 2019; Esenowo et al. 2017; Iqbal et al. 2017).

In contrast to the Pasur, Kohelia, and Karatoya Rivers, where significant spatial variation in phytoplankton assemblages has been documented (Hasan et al. 2022; Mamun et al. 2019; Haque et al. 2021), the Dakatia River exhibited pronounced seasonal but limited spatial dissimilarity among its eight stations, despite distinct physicochemical gradients. This pattern likely reflects the relatively short inter‐station distances and the widespread influence of anthropogenic stressors, resulting in a more homogeneous phytoplankton community structure compared with larger or less anthropogenically impacted river systems. Such contrasts emphasize the importance of river‐specific management approaches and highlight the Dakatia as a valuable case for understanding phytoplankton dynamics under intensive cage culture and mixed industrial pressures. Cage‐culture operations contribute organic matter and dissolved nutrients, especially in the mid‐reaches, while industrial and municipal discharges add metals and additional nutrient loads. During low‐flow periods these inputs accumulate and may select for tolerant, fast‐growing taxa, whereas high monsoon flows redistribute nutrients and dilute point sources, allowing more diverse assemblages to develop. This dynamic mirrors patterns reported in the Pasur and Karatoya Rivers but with distinct seasonal signatures due to the Dakatia's smaller size and intensive aquaculture footprint. Understanding these mechanisms is crucial for managing water quality and aquaculture sustainability. The significant increase in the biomass of green algae and euglenoids during the monsoon months suggests an opportunistic bloom response to nutrient enrichments, especially ammonium introduced through runoff and freshwater discharges from aquaculture (Hasan et al. 2022; Sarker et al. 2021). Dominance by cyanobacteria during summer coincided with high temperatures, weak flow rates, and low nutrient levels, consistent with patterns observed in other subtropical river systems where blooms occur under environmental stress (Haque et al. 2021; Gogoi et al. 2019). Although cyanobacteria are relatively minor components at other times, their sporadic presence during hot and saline months suggests that community structure could shift with increasing salinity intrusion or other environmental changes. In addition to temperature, flow, and nutrient levels, multiple factors—including grazing pressure, light availability, hydrological variability, sedimentation, turbidity, and anthropogenic disturbances—likely interact to shape seasonal community dynamics. Overall, our results suggest that the phytoplankton community of the Dakatia River is shaped by the combined effects of anthropogenic inputs and hydrological seasonality rather than by single stressors alone. These results confirm that the interplay between seasonal hydrology, nutrient cycling, and both biotic and abiotic interactions regulates community composition, with important implications for food web stability and freshwater management in the Dakatia basin. Our findings underscore that monitoring total nutrients alone is insufficient; seasonal shifts in nutrient speciation, hydrological connectivity, and ecological pressures can fundamentally alter community responses. Effective management strategies should therefore account for temporal and spatial variability as well as multiple confounding ecological and environmental factors when designing monitoring programs or establishing nutrient discharge limits.

Although the present study contributes substantially to the understanding of phytoplankton community structure in a subtropical coastal river, several limitations should be acknowledged when interpreting the results. First, correlations between environmental variables and phytoplankton taxa do not necessarily indicate direct causal relationships, as many variables covary seasonally (e.g., nutrient inflow and turbidity) and may collectively influence community structure. Second, the use of a 25 μm mesh size in sampling primarily captured the larger phytoplankton fraction; consequently, smaller picophytoplankton and nanoplankton were likely underrepresented. These groups can substantially contribute to primary production but were beyond the scope of the present study. Finally, single‐day triplicate sampling per station in each season provides a seasonal snapshot rather than continuous time‐series; long‐term monitoring would better resolve transient blooms and short‐term perturbations. We therefore interpret the reported relationships as strong indicators of association that warrant follow‐up with higher‐frequency sampling and complementary methods (e.g., flow cytometry or molecular assays) where possible.

## 5 Conclusion

This study provides the first comprehensive account of phytoplankton diversity and environmental drivers in the Dakatia River, documenting 37 genera across six major classes, with 13 genera consistently present throughout the year and nine dominating the community. The moderate yet stable diversity indices highlight the river's ecological suitability for sustaining primary productivity and supporting fisheries. Seasonal dynamics were found to be a stronger determinant of community structure than spatial variation. ANOSIM confirmed significant seasonal dissimilarities (*p* < 0.001), while no clear spatial dissimilarities were detected (*p* > 0.05). Cluster analysis further delineated five major assemblages, and SIMPER analysis identified *Melosira* sp. as the primary contributor to inter‐seasonal variation, underscoring its ecological importance. These findings demonstrate that the community is not randomly assembled but shaped by hydrological and nutrient regimes.

Environmental factors played a decisive role in regulating phytoplankton abundance and diversity. Correlation analyses highlighted ammonia, temperature, salinity, nitrate, and phosphate as key drivers, with their seasonal fluctuations strongly influencing the composition and abundance of different groups. For example, diatoms thrived in cooler, nutrient‐stable winter waters dominated by nitrate and phosphate, while monsoon runoff enriched with ammonium supported opportunistic green algae and euglenoids. In contrast, cyanobacteria prevailed in nutrient‐poor summer conditions, reflecting their ability to tolerate higher temperatures and low flow, while sporadic dinoflagellate presence suggested potential sensitivity to salinity shifts. Collectively, these results reveal the dynamic interplay of nutrients, hydrology, and phytoplankton succession in a subtropical river system.

Beyond describing the observed patterns, this study provides essential baseline data for the Dakatia River, an ecosystem experiencing increasing anthropogenic pressure from aquaculture expansion, industrial effluents, and agricultural runoff. The findings stressed the sensitivity of primary producers to seasonal environmental changes, reinforcing their role as sentinels of ecosystem health. Importantly, this baseline understanding offers valuable guidance for sustainable water resource management, biodiversity conservation, and the development of aquaculture practices that safeguard both ecological balance and seafood safety.

## Author Contributions

**Md. Saeduzzaman Faraji:** data curation (lead), formal analysis (lead), investigation (lead), writing – original draft (equal). **Md. Mofizur Rahman:** conceptualization (lead), writing – original draft (equal). **Md. Milon Sarker:** formal analysis (equal), investigation (equal), writing – original draft (equal). **Mehedi Mahmudul Hasan:** resources (supporting), writing – review and editing (supporting). **Asma Jaman:** data curation (supporting), formal analysis (supporting), investigation (supporting), resources (equal). **Hea Ja Baek:** resources (supporting), software (supporting), writing – review and editing (supporting). **Takaomi Arai:** resources (lead), writing – review and editing (supporting). **Norhayati Ngah:** resources (supporting), software (supporting), writing – review and editing (supporting). **M. Belal Hossain:** resources (supporting), software (supporting), writing – original draft (supporting), writing – review and editing (lead).

## Conflicts of Interest

The authors declare no conflicts of interest.

## Supporting information


**Appendix S1:** ece372787‐sup‐0001‐AppendixS1.zip.

## Data Availability

Data are provided in the article and in Supporting Information.
