# Supplementary material for: Diversity, Community Assemblage, and Environmental Determinants of Phytoplankton in a Subtropical Transboundary Coastal River
Source: Ecol Evol. 2025 Dec 23;15(12):e72787. doi: 10.1002/ece3.72787 (PMC12723322; doi:10.1002/ece3.72787)
Supplement: Supplementary file 1 — Appendix S1: ece372787‐sup‐0001‐AppendixS1.zip. [file ECE3-15-e72787-s001.zip › Supplementary files (24.11.25).docx]

**Supplementary 1** Phytoplankton genera (sp.) recorded from the Dakatia river, Chandpur, Bangladesh.

|  | S | M | W |  | S | M | W |
| --- | --- | --- | --- | --- | --- | --- | --- |
| Cyanophyceae |  |  |  | *Ankistrodesmus* ^a^ | + | + | + |
| *Anabaena* | - | + | - | *Ceratium* ^a^ | + | + | - |
| *Aphanocapsa* | - | + | + | *Chlorella* ^abc^ | + | + | + |
| *Aphanothece* ^abc^ | + | + | + | *Chlorogonium* ^b^ | + | + | - |
| *Gomphosphaeria* ^a^ | + | + | - | *Closterium* ^b^ | - | + | - |
| *Merismopedia* | + | + | - | *Oocystis* | - | + | - |
| *Microcystis* ^c^ | - | + | + | *Pediastrum* ^abc^ | + | + | + |
| *Nostoc* | + | - | + | *Scenedesmus* | - | + | + |
| *Oscillatoria* ^b^ | - | + | - | *Sphaerocystis* ^abc^ | + | + | + |
| Bacillariophyceae |  |  |  | *Tetraedron* ^ab^ | + | + | - |
| *Amphora* | - | + | + | *Volvox* ^ab^ | + | + | + |
| *Cosmarium* | - | + | - | **Euglenophyceae** |  |  |  |
| *Cyclotella* ^c^ | + | + | + | *Euglena* ^abc^ | + | + | + |
| *Cymbella* | + | - | - | *Phacus* ^ab^ | + | + | - |
| *Fragilaria* ^abc^ | + | + | + | *Trachelomonas* ^bc^ | - | + | + |
| *Melosira* ^abc^ | + | + | + | **Ulvophyceae** |  |  |  |
| *Navicula* | + | - | - | *Ulothrix* ^abc^ | + | + | + |
| *Rhizosolenia* | - | - | + | **Zynematophyceae** |  |  |  |
| *Synedra* ^abc^ | + | + | + | *Micrasterius* ^c^ | - | - | + |
| *Thalassiothrix* ^c^ | - | - | + | *Prorocentrum* ^c^ | **-** | **-** | **+** |
| Cholorophyceae |  |  |  | *Zygnema* ^a^ | + | + | - |
| *Actinastrum* ^c^ | + | + | + |  |  |  |  |

Note: ‘S’, summer; ‘M’, monsoon; ‘W’, winter; ‘a’, dominant in summer; ‘b’, dominant in monsoon; ‘c’, dominant in winter; ‘ab’, dominant in summer and monsoon; ‘bc’, dominant in monsoon and winter; ‘abc’, dominant in all seasons; ‘+’, present; ‘-’, absent.

**Supplementary 2** Pearson's correlation coefficient among physicochemical parameters, phytoplankton abundance, and diversity indices during (A) summer, (B) monsoon, and (C) winter.

|  | Tem | TR | DO | pH | TDS | EC | Sal | PO_4_ | N0_3_ | NH_3_ | Chl | Bac | Cya | Eug | Ulv | Zyg | TP | *H*' | *J*' | *d* |
| --- | --- | --- | --- | --- | --- | --- | --- | --- | --- | --- | --- | --- | --- | --- | --- | --- | --- | --- | --- | --- |
| Tem | 1.000 |  |  |  |  |  |  |  |  |  |  |  |  |  |  |  |  |  |  |  |
| TR | -0.708 | 1.000 |  |  |  |  |  |  |  |  | **A** |  |  |  |  |  |  |  |  |  |
| DO | -0.717 | 0.553 | 1.000 |  |  |  |  |  |  |  |  |  |  |  |  |  |  |  |  |  |
| pH | -0.007 | 0.243 | 0.538 | 1.000 |  |  |  |  |  |  |  |  |  |  |  |  |  |  |  |  |
| TDS | 0.533 | -0.634 | 0.045 | 0.436 | 1.000 |  |  |  |  |  |  |  |  |  |  |  |  |  |  |  |
| EC | 0.405 | -0.679 | -0.018 | 0.313 | 0.909 | 1.000 |  |  |  |  |  |  |  |  |  |  |  |  |  |  |
| Sal | -0.751 | 0.614 | 0.577 | 0.380 | -0.191 | -0.033 | 1.000 |  |  |  |  |  |  |  |  |  |  |  |  |  |
| PO_4_ | 0.729 | -0.652 | -0.260 | 0.304 | 0.810 | 0.599 | -0.401 | 1.000 |  |  |  |  |  |  |  |  |  |  |  |  |
| N0_3_ | 0.563 | -0.664 | -0.007 | 0.314 | 0.911 | 0.725 | -0.363 | 0.926 | 1.000 |  |  |  |  |  |  |  |  |  |  |  |
| NH_3_ | 0.255 | -0.224 | -0.645 | -0.749 | -0.237 | -0.048 | -0.360 | -0.289 | -0.322 | 1.000 |  |  |  |  |  |  |  |  |  |  |
| Chl | -0.649 | 0.633 | **0.688** | 0.509 | -0.023 | 0.108 | **0.872** | -0.401 | -0.271 | -0.238 | 1.000 |  |  |  |  |  |  |  |  |  |
| Bac | -0.069 | -0.389 | 0.304 | 0.262 | 0.415 | 0.381 | 0.078 | 0.355 | 0.453 | -0.661 | -0.145 | 1.000 |  |  |  |  |  |  |  |  |
| Cya | -0.688 | 0.631 | 0.633 | 0.557 | -0.309 | -0.271 | **0.784** | -0.312 | -0.319 | -0.723 | 0.592 | 0.295 | 1.000 |  |  |  |  |  |  |  |
| Eug | **0.777** | -0.295 | -0.671 | -0.180 | 0.172 | 0.079 | -0.648 | 0.334 | 0.186 | 0.599 | -0.398 | -0.670 | -0.737 | 1.000 |  |  |  |  |  |  |
| Ulv | 0.490 | -0.666 | -0.287 | -0.274 | 0.354 | 0.263 | -0.675 | 0.317 | 0.367 | 0.096 | -0.678 | 0.491 | -0.577 | 0.088 | 1.000 |  |  |  |  |  |
| Zyg | 0.634 | -0.579 | -0.080 | 0.468 | **0.861** | 0.689 | -0.267 | **0.812** | **0.786** | -0.365 | -0.222 | 0.543 | -0.240 | 0.137 | 0.561 | 1.000 |  |  |  |  |
| TP | -0.158 | 0.259 | 0.572 | 0.944 | 0.414 | 0.372 | 0.595 | 0.205 | 0.223 | **-0.735** | 0.638 | 0.370 | 0.643 | -0.375 | -0.281 | 0.459 | 1.000 |  |  |  |
| *H*' | -0.552 | 0.602 | 0.337 | 0.352 | -0.285 | -0.241 | ***0.841*** | -0.162 | -0.273 | -0.438 | 0.600 | -0.017 | 0.800 | -0.457 | -0.803 | -0.289 | 0.477 | 1.000 |  |  |
| *J*' | -0.098 | -0.023 | 0.363 | 0.754 | 0.468 | 0.492 | 0.588 | 0.369 | 0.345 | -0.727 | 0.442 | 0.599 | 0.626 | -0.492 | -0.201 | 0.512 | 0.880 | 0.555 | 1.000 |  |
| *d* | -0.584 | **0.766** | 0.269 | 0.143 | -0.508 | -0.500 | **0.739** | -0.338 | -0.463 | -0.213 | 0.552 | -0.314 | 0.652 | -0.283 | -0.848 | -0.510 | 0.231 | 0.926 | 0.221 | 1.00 |
|  | **Tem** | **TR** | **DO** | **pH** | **TDS** | **EC** | **Sal** | **PO_4_** | **N0_3_** | **NH_3_** | **Chl** | **Bac** | **Cya** | **Eug** | **Ulv** | **Zyg** | **TP** | ***H*'** | ***J*'** | ***d*** |
| Tem | 1.000 |  |  |  |  |  |  |  |  |  | **B** |  |  |  |  |  |  |  |  |  |
| TR | 0.761 | 1.000 |  |  |  |  |  |  |  |  |  |  |  |  |  |  |  |  |  |  |
| DO | -0.651 | -0.925 | 1.000 |  |  |  |  |  |  |  |  |  |  |  |  |  |  |  |  |  |
| pH | -0.759 | -0.886 | 0.812 | 1.000 |  |  |  |  |  |  |  |  |  |  |  |  |  |  |  |  |
| TDS | -0.679 | -0.822 | 0.880 | 0.865 | 1.000 |  |  |  |  |  |  |  |  |  |  |  |  |  |  |  |
| EC | -0.722 | -0.946 | 0.954 | 0.848 | 0.770 | 1.000 |  |  |  |  |  |  |  |  |  |  |  |  |  |  |
| Sal | -0.774 | -0.845 | 0.870 | 0.865 | 0.923 | 0.837 | 1.000 |  |  |  |  |  |  |  |  |  |  |  |  |  |
| PO_4_ | -0.668 | -0.865 | 0.950 | 0.794 | 0.957 | 0.849 | 0.939 | 1.000 |  |  |  |  |  |  |  |  |  |  |  |  |
| N0_3_ | -0.571 | -0.797 | 0.905 | 0.679 | 0.925 | 0.758 | 0.872 | 0.975 | 1.000 |  |  |  |  |  |  |  |  |  |  |  |
| NH_3_ | -0.763 | -0.792 | 0.656 | 0.956 | 0.758 | 0.735 | 0.833 | 0.674 | 0.538 | 1.000 |  |  |  |  |  |  |  |  |  |  |
| Chl | 0.451 | 0.455 | -0.404 | -0.666 | -0.638 | -0.338 | -0.380 | -0.427 | -0.380 | -0.560 | 1.000 |  |  |  |  |  |  |  |  |  |
| Bac | -0.339 | -0.542 | 0.700 | 0.252 | 0.613 | 0.512 | 0.491 | **0.730** | ***0.828*** | 0.040 | -0.189 | 1.000 |  |  |  |  |  |  |  |  |
| Cya | 0.235 | -0.109 | 0.220 | 0.051 | 0.178 | 0.077 | -0.163 | 0.132 | 0.176 | -0.189 | -0.528 | 0.402 | 1.000 |  |  |  |  |  |  |  |
| Eug | -0.456 | -0.032 | -0.071 | -0.060 | 0.020 | -0.066 | 0.039 | -0.014 | 0.066 | -0.042 | -0.073 | 0.223 | -0.315 | 1.000 |  |  |  |  |  |  |
| Ulv | -0.506 | -0.490 | 0.397 | 0.698 | 0.673 | 0.369 | 0.698 | 0.570 | 0.469 | **0.799** | -0.480 | 0.004 | -0.162 | -0.216 | 1.000 |  |  |  |  |  |
| Zyg | -0.683 | **-0.744** | 0.543 | ***0.885*** | 0.544 | 0.695 | 0.596 | 0.458 | 0.294 | ***0.913*** | -0.584 | -0.116 | -0.033 | -0.104 | 0.608 | 1.000 |  |  |  |  |
| TP | -0.037 | -0.258 | 0.422 | -0.153 | 0.195 | 0.280 | 0.200 | 0.423 | 0.543 | -0.305 | 0.355 | 0.844 | 0.167 | 0.137 | -0.284 | -0.431 | 1.000 |  |  |  |
| *H*' | **-0.779** | -0.539 | 0.367 | 0.703 | 0.643 | 0.380 | 0.666 | 0.521 | 0.426 | **0.801** | -0.594 | 0.065 | -0.219 | 0.237 | 0.869 | 0.665 | -0.289 | 1.000 |  |  |
| *J*' | -0.165 | -0.324 | 0.192 | 0.252 | -0.157 | 0.449 | 0.091 | -0.045 | -0.171 | 0.304 | 0.304 | -0.328 | -0.322 | -0.219 | -0.138 | 0.501 | -0.143 | -0.157 | 1.000 |  |
| *d* | **-0.756** | -0.461 | 0.324 | 0.610 | 0.609 | 0.318 | 0.655 | 0.511 | 0.425 | **0.720** | -0.492 | 0.097 | -0.283 | 0.254 | 0.860 | 0.543 | -0.210 | 0.983 | -0.236 | 1.000 |
|  | **Tem** | **TR** | **DO** | **pH** | **TDS** | **EC** | **Sal** | **PO_4_** | **N0_3_** | **NH_3_** | **Chl** | **Bac** | **Cya** | **Eug** | **Ulv** | **Zyg** | **TP** | ***H*'** | ***J*'** | ***d*** |
| Tem | 1.000 |  |  |  |  |  |  |  |  |  |  |  |  |  |  |  |  |  |  |  |
| TR | -0.265 | 1.000 |  |  |  |  |  |  |  |  | **C** |  |  |  |  |  |  |  |  |  |
| DO | -0.125 | 0.320 | 1.000 |  |  |  |  |  |  |  |  |  |  |  |  |  |  |  |  |  |
| pH | -0.159 | 0.495 | 0.932 | 1.000 |  |  |  |  |  |  |  |  |  |  |  |  |  |  |  |  |
| TDS | 0.474 | 0.121 | 0.538 | 0.362 | 1.000 |  |  |  |  |  |  |  |  |  |  |  |  |  |  |  |
| EC | 0.587 | -0.467 | -0.232 | -0.342 | 0.517 | 1.000 |  |  |  |  |  |  |  |  |  |  |  |  |  |  |
| Sal | -0.621 | 0.085 | 0.706 | 0.644 | 0.089 | -0.287 | 1.000 |  |  |  |  |  |  |  |  |  |  |  |  |  |
| PO_4_ | 0.521 | 0.418 | 0.491 | 0.483 | 0.513 | -0.216 | -0.164 | 1.000 |  |  |  |  |  |  |  |  |  |  |  |  |
| N0_3_ | 0.101 | 0.501 | 0.907 | 0.885 | 0.606 | -0.195 | 0.504 | 0.709 | 1.000 |  |  |  |  |  |  |  |  |  |  |  |
| NH_3_ | 0.599 | -0.230 | -0.559 | -0.555 | 0.005 | 0.239 | -0.904 | 0.225 | -0.480 | 1.000 |  |  |  |  |  |  |  |  |  |  |
| Chl | -0.298 | -0.312 | 0.275 | 0.084 | 0.111 | -0.154 | 0.607 | -0.071 | 0.145 | -0.389 | 1.000 |  |  |  |  |  |  |  |  |  |
| Bac | -0.312 | -0.144 | 0.466 | 0.381 | 0.063 | -0.351 | 0.680 | 0.097 | 0.324 | -0.413 | 0.910 | 1.000 |  |  |  |  |  |  |  |  |
| Cya | -0.458 | -0.089 | 0.268 | 0.125 | 0.088 | -0.252 | 0.626 | -0.101 | 0.141 | -0.417 | 0.956 | 0.893 | 1.000 |  |  |  |  |  |  |  |
| Eug | 0.172 | 0.071 | 0.313 | 0.465 | -0.064 | -0.336 | -0.072 | 0.427 | 0.213 | 0.318 | -0.188 | 0.183 | -0.190 | 1.000 |  |  |  |  |  |  |
| Ulv | -0.464 | 0.362 | 0.404 | 0.545 | 0.053 | 0.030 | 0.670 | -0.328 | 0.342 | **-0.787** | 0.010 | 0.130 | 0.125 | -0.127 | 1.000 |  |  |  |  |  |
| Zyg | -0.281 | -0.367 | 0.419 | 0.262 | 0.120 | -0.174 | 0.652 | -0.040 | 0.190 | -0.329 | 0.924 | 0.952 | 0.873 | 0.146 | 0.052 | 1.000 |  |  |  |  |
| TP | -0.332 | -0.151 | 0.470 | 0.383 | 0.073 | -0.332 | 0.692 | 0.067 | 0.310 | -0.414 | 0.908 | 0.998 | 0.898 | 0.188 | 0.151 | 0.961 | 1.000 |  |  |  |
| *H*' | 0.289 | -0.461 | 0.423 | 0.191 | 0.450 | 0.408 | 0.176 | 0.078 | 0.263 | -0.088 | -0.028 | -0.115 | -0.229 | -0.004 | -0.090 | 0.090 | -0.105 | 1.000 |  |  |
| *J*' | 0.305 | -0.149 | 0.222 | 0.098 | 0.380 | 0.365 | -0.199 | 0.119 | 0.104 | 0.236 | -0.519 | -0.551 | -0.611 | 0.142 | -0.163 | -0.359 | -0.531 | 0.811 | 1.000 |  |
| *d* | 0.019 | -0.281 | 0.631 | 0.432 | 0.406 | 0.230 | 0.623 | 0.028 | 0.525 | -0.620 | 0.302 | 0.228 | 0.129 | -0.230 | 0.309 | 0.316 | 0.227 | 0.800 | 0.384 | 1.000 |

Abbreviations: Tem, temperature; TR, transparency; DO, dissolved oxygen; TDS, total dissolved solids; EC, electrical conductivity; Sal, salinity; PO_4_, phosphate; NO_3_, nitrate; NH_3_, ammonia; Chl, Chlorophyceae; Bac, Bacillariophyceae; Cya, Cyanophyceae; Eug, Euglenophyceae; Ulv, Ulvophyceae; Zyg, Zygnematophyceae; TP, total phytoplankton; *H*', Shanon-Wiener diversity index; *J*', species evenness; *d*, species richness

Bold, significantly correlated at *p* < 0.05; Bold and italic, significantly correlated at *p* < 0.01
